# Limpet II: A Modular, Untethered Soft Robot

**DOI:** 10.1089/soro.2019.0161

**Published:** 2021-06-16

**Authors:** Mohammed E. Sayed, Jamie O. Roberts, Ross M. McKenzie, Simona Aracri, Anthony Buchoux, Adam A. Stokes

**Affiliations:** ^1^ Institute for Integrated Micro and Nano Systems, Scottish Microelectronics Centre, School of Engineering, The University of Edinburgh, Edinburgh, United Kingdom.; ^2^ Engineering and Physical Sciences Research Council (EPSRC) Centre for Doctoral Training (CDT) in Robotics and Autonomous Systems, School of Informatics, The University of Edinburgh, Edinburgh, United Kingdom.

**Keywords:** modular, robot, soft, climbing, electromagnetic actuator, adhesion

## Abstract

The ability to navigate complex unstructured environments and carry out inspection tasks requires robots to be capable of climbing inclined surfaces and to be equipped with a sensor payload. These features are desirable for robots that are used to inspect and monitor offshore energy platforms. Existing climbing robots mostly use rigid actuators, and robots that use soft actuators are not fully untethered yet. Another major problem with current climbing robots is that they are not built in a modular fashion, which makes it harder to adapt the system to new tasks, to repair the system, and to replace and reconfigure modules. This work presents a 450 g and a 250 × 250 × 140 mm modular, untethered hybrid hard/soft robot—Limpet II. The Limpet II uses a hybrid electromagnetic module as its core module to allow adhesion and locomotion capabilities. The adhesion capability is based on negative pressure adhesion utilizing suction cups. The locomotion capability is based on slip-stick locomotion. The Limpet II also has a sensor payload with nine different sensing modalities, which can be used to inspect and monitor offshore structures and the conditions surrounding them. Since the Limpet II is designed as a modular system, the modules can be reconfigured to achieve multiple tasks. To demonstrate its potential for inspection of offshore platforms, we show that the Limpet II is capable of responding to different sensory inputs, repositioning itself within its environment, adhering to structures made of different materials, and climbing inclined surfaces.

## Introduction

### Offshore environment requirements

Offshore platforms are massively complex and unstructured environments. The environment consists of structures at different angles, steep inclines, and inverted surfaces. To navigate such unstructured environments efficiently and to be useful for inspection and exploration tasks, a robot must have a good adhesion mechanism and be capable of multiple locomotion modalities such as walking, running, and climbing.^1^ The ability to adhere and to climb these inclined surfaces increases the accessible areas of terrestrial robots in such environments, which improves the capability of the robot to do inspection and monitoring of the offshore platform. Adhering to and climbing on these unstructured offshore environments alone is not enough for the robot to carry out inspection tasks; it must also be equipped with a sensor payload.

### Modularity, stacking, and reconfigurability

Robots performing tasks in unstructured environments (e.g., offshore) need to adapt to variable constraints in their environment. Traditional robotics provide unique solutions to a specific real-world application, but they are usually hard to use in other applications due to the lack of their adaptive nature.^2^ Modular robots or assemblies of modular units^3–14^ are easier to adapt to new tasks and make it easier to repair the system, to replace modules, and to control the robot. Modular robots are usually made of some primary structural actuated units and some additional specialized units (e.g., grippers, sensor payload, feet, etc.).^15^ The interest in modular systems is due to the hypothesis that a single, advanced robot is more expensive and less robust than multiple low-cost modules.^2,15,16^

Modular robots can be easily reconfigured for different tasks,^2,3,6,15,17–19^ making them different from monolithic robots. Modular robots provide the advantage of: reconfigurability, reusability, versatility, low cost, robustness, and ease of manufacturing over traditional robotic systems.^2,20^ The capability of a modular robotic system is usually dependent on the number of modules within it.^15^ However, the number of modules is often limited by the cost of the unit, where systems with large number of modules sacrifice their functionality to keep the costs low. Creating low-cost modular robotic systems with higher capabilities is a key challenge in modular robotics. The use of hardware for multifunctional purposes is a great way to keep costs of the system low. Examples include the Kilobot system^21^ and the Hoverbot system.^22,23^

The capability of single modules is limited, but modular robots consisting of multiple units have increased capability compared with the sum of their parts.^24^ By stacking modules, we can increase the overall capability and the range of behaviors and motions of a system, and, thus, utilize it to perform useful tasks. The term “stacking” refers to the ability to combine functional units to develop a system that has greater capability and complexity than the sum of its individual units.^24^ There are multiple examples of systems where higher capability was achieved by stacking functional modules together.^25–47^

### Climbing robots

Climbing robots can be used for a wide range of applications in unstructured environments (e.g., offshore). The two key features for climbing robots are adhesion and locomotion mechanisms. Recent works on climbing robots have adapted different bio-inspired and engineered adhesion mechanisms. The adhesion mechanisms include gecko-inspired adhesion (via long-range molecular forces),^48–50^ magnetic adhesion,^51–56^ electroadhesion,^1,57–63^ adhesive materials,^64^ and negative pressure adhesion.^65,66^

Geckos adhere to surfaces via Van der Waals forces by using a large number of very fine hair structures.^67^ Gecko-inspired adhesion has been utilized by climbing robots of different scales to move on smooth inclined surfaces.^68–74^ Magnetic adhesion relies on generating a magnetic force by using permanent magnets, electromagnets, or electro-permanent magnets. Magnetic adhesion has the advantage of facile control, but it works only on ferromagnetic surfaces. Electroadhesion is achieved by using an applied electric field, and it can be tuned by changing the input voltage modulation.^1^ Electroadhesion requires a large input voltage, and it also uses power tethers.^1^ Adhesive materials for climbing robots are not very reliable and they require smooth and clean surfaces.^64^

The most widely adopted adhesion mechanism for climbing robots is negative pressure adhesion using suction cups. This adhesion method has higher reliability, simpler design, and easier application than other adhesion methods. Adhesion in nature based on suction can be seen in many different animals, including northern clingfish,^75^ limpet,^76^ octopus,^77,78^ and squid.^79^ The suction mechanism demonstrates effectiveness in both terrestrial^30,80^ and aquatic environments.^81^ Suction mechanisms have also been applied by robots in other applications, such as picking objects with variable shape and size.^82^ It is important to have sensory feedback on the adhesion module, to inform the status of adhesion of the system and to implement closed-loop control systems that can enable robots to maintain their adhesion for longer periods.

Locomotion types of climbing robots include crawlers,^83–86^ wheels,^87,88^ and legs.^1,89–95^ Crawler-type climbing robots can move relatively fast, but they are not adequate for rough environments as they cannot cross obstacles easily. The most common strategy for locomotion is using a two-wheel differential drive, where each wheel is powered by an electric motor. Wheeled robots are capable of achieving high velocities, do not need actuator calibration, and are platform independent. Wheeled locomotion, however, has a negative impact on the battery life and the cost of the robot. Another main disadvantage of using wheeled locomotion for robots that use suction force adhesion is the need to maintain an air gap between the robot base and the surface the robot is moving on. This feature will create some problems for the robot-like loss of pressure or friction with the surface.^96^ Legged robots allow the creation of a stable and strong adhesion force to the surface, and they can cope well with cracks or obstacles in their environment.^96^ The disadvantage of legged robots is that they achieve low speeds and may require complex control. There have been various other climbing robots based on different adhesion and locomotion mechanisms reported recently.^64,97–105^

### The Limpet II

In this work, we introduce our modular, untethered hybrid robot—Limpet II—which uses a multifunctional electromagnetic module (EMM) and a sensing module to achieve increased capability and reconfigurability while keeping the costs low. The modularity of the Limpet II allows it to climb inclined surfaces, adhere to different structures, and sense and respond to changes in its environment. Supplementary Video S1 shows an overview of the Limpet II system and its capabilities. The Limpet II is being developed as part of the Offshore Robotics for Certification of Assets (ORCA Hub) in the United Kingdom. The ORCA Hub is a 3.5 year Engineering and Physical Sciences Research Council (EPSRC) funded multisite project aiming at using teams of robots and autonomous intelligent systems on remote energy platforms to enable cheaper, safer, and more efficient working practices.^106^ Sensing is a key component to the ORCA Hub, as it is important for inspection, monitoring structural and asset health, fault detection, environmental monitoring, mapping, and helping other robots navigate around the platform. The Limpet II comprises the sensing aspect of the ORCA Hub.

We based our untethered modular robot presented in this article on version 1 of the Limpet^107^ and the Linbot system.^108^ We developed version 1 of the Limpet as a multi-sensing platform for monitoring offshore platforms. We developed the Linbots to show a collective of low-cost robots that can be reconfigured to achieve different useful tasks. The Linbot is a soft, modular robot that uses a multi-functional EMM for linear actuation, communication, sound output, and tactile sensing. The Linbot and the Limpet version 1 are not capable of locomotion or adhesion. In this article, we define the Limpet version 1 as the sensing module. The EMM used for the Linbot system is utilized in this article to achieve locomotion and adhesion for Limpet II. The following sections describe the design and evaluation of Limpet II and all its modules.

The Limpet II is inspired by patella vulgata,^109^ commonly known as limpet, which is an aquatic snail with a conical shell that lives on rocks on land or in the sea. The patella vulgata has a strong shell that protects it from the external environment and predators. It can alternate between different adhesion mechanisms depending on the required period and strength of adhesion.^76^ Suction is the primary adhesion mechanism used by patella vulgata, where the suction force can reach up to 25 N.^110^ The shell of the patella vulgata also plays an important role in the adhesion mechanism. The patella vulgata clamps its shell to increase friction with the surface and to prevent any dislodgements caused by shear forces.^110^ Shell clamping against the surface makes use of the vertical adherence strength of the suction mechanism to create a frictional force that resists the shear force of hydrodynamic drag.^110^ The patella vulgata has an optic vesicle at the end of its tentacles that provides sensory function, and enables it to sense light and darkness levels.^109^

### Objectives

Our objective in this work was to design a hybrid robot in a modular fashion—Limpet II—so that the modules can be reconfigured to achieve different tasks such as climbing inclined surfaces, adhering to structures, and sensing its environment. The primary purpose of this robot is to inspect and monitor offshore energy platforms and, as such, these tasks are vital to allow the robot to be useful in its environment. The robot consists of a sensing module, power driver module, and adhesion and locomotion modules based on EMMs. We explained the design procedure and fabrication techniques for each of the different modules in the Limpet II system. We described the experimental methods used to characterize the performance of each of the different modules. It is important to characterize each of the modules in a modular robot to understand the capabilities of the single modules and how they can come together to create a more complex and useful system. Finally, we demonstrated the capability of the final Limpet II system in climbing inclined surfaces, adhering to structures, and responding to sensory input by reconfiguring the modules of the system.

## Materials and Methods

### System design

The Limpet II uses a low-cost and manufacturable EMM as its core module. The EMM can actuate linearly, and it can produce sound output. We used the EMM to construct adhesion and locomotion modules, to give the Limpet II the capability of locomotion, adhesion, and sound communication. The Limpet II is designed as a modular system with multi-sensing capabilities. The modular architecture allows the Limpet II to achieve multiple complex tasks in its environments, including climbing, adhesion, and structural health monitoring. The Limpet II is a hybrid system combining between hard and soft materials. Hybrid systems can take advantage of rigid components while still being able to partially conform to their environment.^33^ The soft materials in the Limpet II system give it the advantage of inherent compliance, low-cost manufacturing, and improved safety. The soft material compliance can immensely help robots in unstructured environments adapt to unexpected environmental changes such as slopes and obstacles. We have developed the Limpet II as an untethered system. The dependence on electrical or pneumatic tethers limits the applications that can be performed by robots in their environment.^42,111^ For systems to be useful in field applications, all their vital components (e.g., actuation, power, communication, and processing components) need to be fully integrated within their structure.^111^ The experiments we conducted to characterize the different modules within the Limpet II used power and pneumatic tethers. However, the final Limpet II system combining all the modules was developed as an untethered system.

**FIG. 1.**
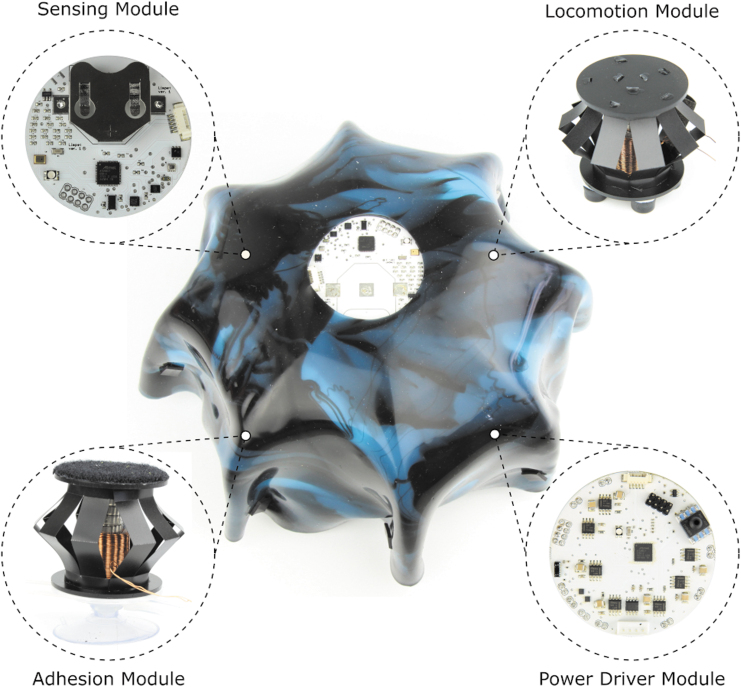
System overview. The Limpet II is a modular, untethered hybrid hard/soft robot consisting of a sensing module, locomotion module, adhesion module, power driver module, and outer soft shell. Color images are available online.

The Limpet II system consists of a sensing module (Limpet version 1), a power driver module, an adhesion module, a locomotion module, and an outer protective shell as shown by Figure 1. The main purpose of the Limpet II is to monitor the offshore assets and the environmental conditions surrounding the assets.^107^ We equipped the Limpet II with nine exteroceptive sensing modalities. The sensing modalities on the Limpet II system are all incorporated on the sensing module. The sensors included on the Limpet II are: temperature, pressure, humidity, optical, distance, sound, magnetic, accelerometer, and gyroscope. Our choice of sensing modalities was based on the ability to measure parameters that are relevant to monitoring the environmental conditions on the offshore platform and monitoring the health of the offshore structures. The choice of sensing modalities was also influenced by discussions and input from industrial partners in the ORCA Hub project. Each sensor on the Limpet II can convert a physical measurement variable into a corresponding signal variable, where the physical measurement variable can be related to multiple measurands (events) giving the Limpet II multi-functional sensing capabilities.^107^ Table 1 gives an example of how each sensor can be used to detect multiple measurands in offshore platforms, and, thus, gives an overview of the multi-functionality that can be achieved with the Limpet II.

**Table 1.** Measurands (Events) from the Sensors on the Limpet II and Some Application Examples

Sensor	Physical measurement variable	Signal variable	Measurands	Application examples
Accelerometer	Acceleration	Acceleration	Inclination, collision, vibration, free-fall detection, movement acceleration	Monitoring conductor motion and vibration; monitoring floating production storage and offloading vessel movement
Gyroscope	Angular velocity	Angular velocity	Tilt detection, orientation	Monitoring the orientation and angle of drilling equipment; monitoring floating production storage and offloading vessel tilt and roll
Temperature	Temperature	Temperature	Ambient temperature, over-heating, fire detection	Overheating of structures and equipment
Humidity	Humidity	Humidity	Relative humidity	Monitoring humidity levels that can cause problems related to corrosion of components and issues to materials used as additives
Microphone	Sound	Voltage	Speech recognition, noise cancellation, audible fault detection	Monitoring vibration of vessels, pipes, structures; Structural health monitoring of platform and structures
Pressure	Pressure	Pressure	Ambient pressure	Flow and pressure measurement in vessels
Hall-effect	Magnetic field	Magnetic flux density	Locating pipelines, corrosion detection	Monitoring corrosion in pipes and wind turbines
Optical	Light (visible)	Light intensity	Ambient light intensity, local communication, color detection	Monitoring the level of debris in the air
Distance (time-of-flight)	Light (laser)	Distance	Fault detection, proximity, collision detection, object identification	Monitoring vibration of structures, pipes, vessels, and conductors; structural Health monitoring of wind turbines

#### Sensing modalities, communication, and programming

The sensors on the sensing module, except for the sound sensor, are controlled by the microcontroller through the I^2^C bus. The sound sensor has an analog output and is connected to an analog-to-digital converter on the microcontroller. We designed the Limpet II to have robust communication. It can transmit data to a PC or other nodes by using one of several different communication systems, including WiFi, serial, LoRa, optical, and infrared (IR) communication. WiFi and serial communication do not allow for communication with neighboring Limpets, unlike LoRa, optical, and IR communication. Serial, WiFi, and IR communication are high-bandwidth communication, and, as such, we can send the sensor data from the Limpet II continuously in real time to the PC. The data can then be analyzed on the PC later. In this regard, the Limpet II is only making a measurement by using the sensors and transmitting it instantly to the PC without doing any processing on the data, which means that serial and Wi-Fi communication are not computationally demanding on the Limpet II. LoRa and optical communication have a much lower communication bandwidth than serial and Wi-Fi communication. Thus, when these communication technologies are used with the Limpet II, their limited bandwidth limits the ability to send the sensor data continuously in real time to the PC. Therefore, if the dataset is large, processing and analysis of the data need to be done on the microcontroller of the Limpet II before transmission to allow for only small payloads of data to be transmitted.^107^ This on-board processing and analysis approach requires higher computational power than serial, Wi-Fi, and IR communication. Further information on the communication methods used with the Limpet II and how each communication is achieved can be found in The Limpet II section in the Supplementary Data. Schematics of the architecture of the WiFi, serial, and LoRa communication are shown by Supplementary Figures S1–S3, respectively. In this work, we use the on-board IR transceiver for robot-to-computer communication, where the data from the Limpet II and the sensors can be sent via IR to the computer. We debugged the Limpet II via IR by using a custom-designed IR handheld device. The custom-designed IR handheld device consists of an IR transceiver module (TFBS4711), a microcontroller (ATSAMD21E16), and a USB-to-UART (Universal Asynchronous Receiver-Transmitter) convertor (CP2102).

#### Cost and dimensions of the Limpet II

Cost and functionality are the most important factors considered when developing robots. These properties have a great effect on the scalability of a system to a large number of agents. Scaling up robot collectives without sacrificing functionality is a challenging problem. Low-cost robots are scalable, but current systems have limited capabilities, which limit the type of useful tasks that can be performed by robots in “real-world” scenarios. Our rationale behind the Limpet II system was to keep the costs as low as possible without sacrificing functionality. The sensing module is an integrated Printed Circuit Board (PCB) that has a diameter of 50 mm, a height of 3 mm and weighs 10 g. The power driver module is a PCB with a diameter of 65 mm, a height of 3 mm and weighs 15 g. The full Limpet II system has a length of 250 mm, a width of 250 mm, and a height of 140 mm. We designed the Limpet II to have a size and weight ideal for ease of fabrication, manufacturing, and assembly. The total cost of the Limpet II system is about £112. The breakdown of the cost of the different components in the Limpet II system is shown in Table 2.

**Table 2.** Cost Summary of the Limpet II and Its Components

Category	Components	Cost (£)
Sensing module	Electronic components	22
Power driver module	Electronic components	47.96
EMM	Coils	1.22
	Magnets	3.08
	Acetate	0.054
	Acrylic	0.5
Adhesion module	EMM	4.854
	Suction cup	0.18
	Silicon tubing	0.8
	Vacuum pump	8.23
	Solenoid valve	2
Locomotion module	EMM	4.854
	Rubber feet ( × 5)	0.773
Limpet	Adhesion module ( × 1)	16.064
	Locomotion module ( × 4)	22.508
	Sensing module ( × 1)	22
	Power driver module ( × 1)	47.96
	Outer body (shell) ( × 1)	3.8
Total cost	Limpet	112.3

EMM, electromagnetic module.

### Electrical design

#### Design of the control electronics

The sensing module is based on the PCB of version 1 of the Limpet.^107^ The sensing module and its components are shown by Figure 1 and Supplementary Figure S4. The schematic of the sensing module is shown by Supplementary Figure S5. The sensing module incorporates all the sensory elements of the Limpet II. The power driver module is connected to the sensing module and is used to control the frequency of the actuation of the EMMs, air pump, and solenoids, as well as sending out the data from the sensing module to the PC through the IR transceiver. A labeled picture and the PCB schematic of the power driver module is shown by Supplementary Figures S6 and S7, respectively. Further information on the design of the sensing module and power driver module PCBs can be found in Design of the Sensing and Power Driver Modules section in Supplementary Data.

#### Power system

The Limpet II is powered by a 450 mAh 11.1 V lithium polymer (Li-Po) battery. The Li-Po battery is a rechargeable battery and has dimensions of 49 × 28.5 × 16 mm. We detach the Li-Po batteries for charging. The Limpet II has a minimum battery life of 26 min and a maximum of 4500 h. The method we used to determine the battery life can be found in the Limpet II Battery Life section in Supplementary Data.

### Mechanical design

#### Design of the EMM

**FIG. 2.**
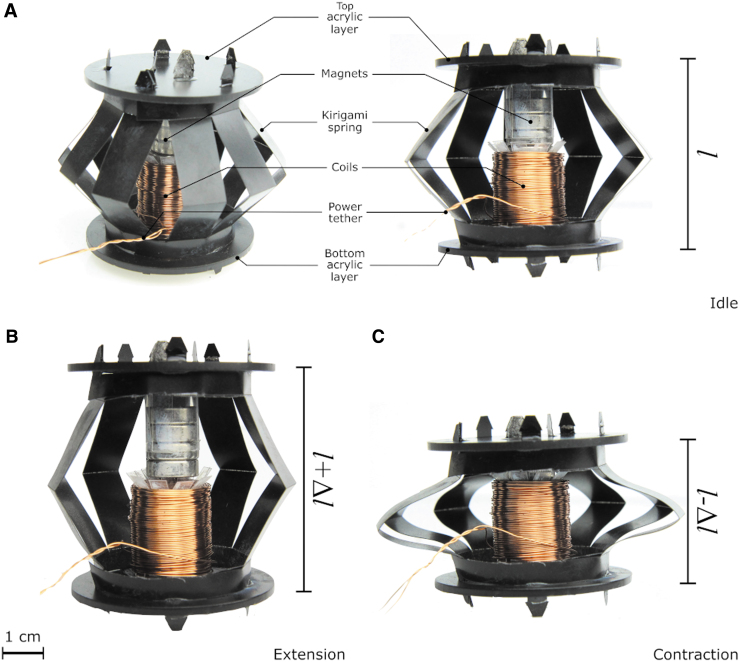
Electromagnetic module. **(A)** A labeled picture of the EMM and a cross-sectional view of the EMM at rest position (l) showing the kirigami spring, coils, magnets, and top and bottom acrylic layers. The coils interact with the magnets and either push or pull the kirigami spring. **(B)** An EMM extending resulting in an increase in length of the module (l + Δl). **(C)** An EMM contracting resulting in a decrease in length of the module (l − Δl). EMM, electromagnetic module. Color images are available online.

The Limpet II is designed as a modular system consisting of an adhesion module, locomotion module, sensing module, and power driver module. The main unit of the Limpet II is the EMM, which is used as the core unit of the adhesion and locomotion modules. Figure 2 shows our EMM. The EMM is an electromagnetic system that is based on the same fundamental principles of loudspeakers. The EMM consists of an actuating electromagnetic coil and permanent magnets, which are attached together by using a soft body that functions as a spring. The electromagnetic coil can repel or attract the permanent magnet depending on the direction of the current applied to the coil, and this force stretches or compresses the body of the robot, creating linear motion. The EMM is a hybrid hard/soft system.

The EMMs have been shown to be a useful system for soft robotics.^44,108^ They are multi-functional components that can generate different behaviors over a range of different control frequencies. At low frequencies, the EMM demonstrates linear actuation capabilities. At audible sound frequencies, the EMM acts as a loudspeaker and at even higher frequencies it can be used for communication,^44,108^ where the communication is based on inductive data transmission, in which the transmission coil generates an alternating magnetic field that induces a voltage in the receiver coil. In this work, we only focus on the actuation and sound capabilities of the EMM and do not utilize its communication capabilities.

Our EMM consists of: electromagnetic coils wound around a reel; permanent magnets embedded in a holder; and a spring consisting of connected bent legs resembling a Chinese lantern as shown by Figure 2A. The magnet holder and the coil reel are attached to the spring by using laser-cut circular acrylic layers. The EMM can be extended or contracted axially from its rest position, depending on the polarity of current applied to the electromagnetic coil in the voice coil system. A sketch of the module and actuation mechanism is shown in Supplementary Figure S8. The internal components of the EMM are shown in Figure 2A. Application of current to the coil allows the actuator unit to be contracted or extended along its central axis from its rest position; as shown by Figure 2B and C. The applied current induces a magnetic field in the coil, which either attracts or repels the permanent magnets, resulting in this actuation mechanism.

#### Design of the adhesion module

**FIG. 3.**
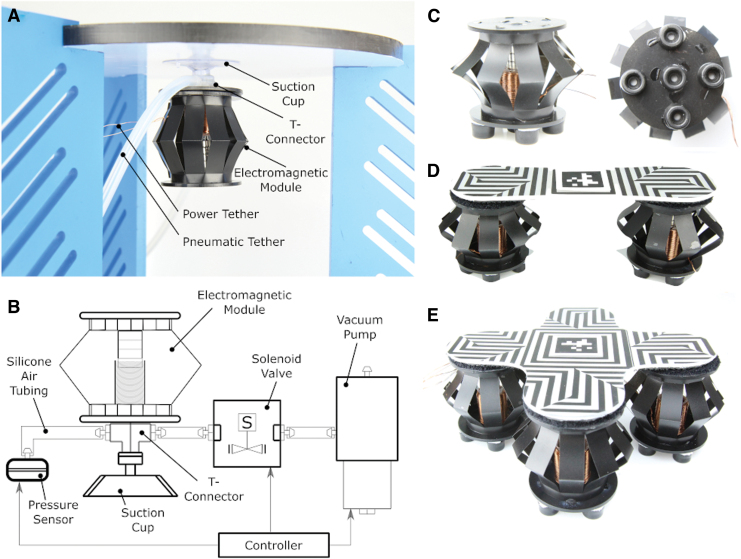
Adhesion module and locomotion modules. **(A)** A labeled picture of the adhesion module showing the EMM, suction cup, T-connector, and pneumatic and power tethers. **(B)** A schematic representation of the adhesion system. **(C)** A side view picture and bottom view picture of the locomotion module based on the EMM. **(D)** A picture of the combination of two locomotion modules based on the EMMs. **(E)** A picture of four EMMs stacked together into a locomotion system. Color images are available online.

To navigate the offshore environment efficiently and be useful for inspection and monitoring of offshore structures, the Limpet II will need to have a good adhesion mechanism that allows robust adhesion to different surfaces. We developed an adhesion module based on the EMM, where the adhesion mechanism is based on negative pressure adhesion. The adhesion module also includes a suction cup, a vacuum pump, a micro solenoid valve, and a pressure sensor. Negative pressure adhesion using suction cups provides the flexibility needed to seal on uneven or coarse surfaces. Our adhesion module is depicted in Figure 3A. Figure 3B shows a schematic representation of the adhesion system. We attach a T-connector to the suction cup. One side of the T-connector is connected to a pressure sensor. The other side of the T-connector is connected to a valve and pump. To attach to a surface, we program the EMM to push initially on the suction cup to flatten the suction cup and push out the air in the cavity between the cup and surface. We then open the valve and turn on the vacuum pump. The pump will remove any remaining air in the cavity and create a vacuum seal. After the seal is created, we close the valve and turn the pump off. To detach the suction cup, we open the solenoid valve to atmosphere, which causes air to rush into the cavity and break the seal. We use a microcontroller (ATSAMD21G18) to control the pressure sensor, solenoid valve, and air pump in the adhesion system. We chose a commercial suction cup for our adhesion module. We compared the commercial suction cup with two different groups of suction cups, which we fabricated out of different soft materials. The two groups of suction cups are: custom-designed suction cups and replicas of the commercial suction cup. We used six different soft materials for each group of suction cups, which are Dragon Skin 10, Dragon Skin 20, Dragon Skin 30, Ecoflex 00-10, Ecoflex 00-30, and Ecoflex 00-50. A picture of the mold used to fabricate the custom-designed suction cups is shown in Supplementary Figure S9. We designed an experimental setup to test the maximum vertical loading capacity and maximum horizontal loading capacity of the suction cups. A picture of the experimental setup and results of the tests can be found in Supplementary Figures S10 and S11, respectively. We conducted the same test for the commercial suction cup, and the results are shown in Supplementary Figure S12. The commercial suction cup can handle a much higher loading capacity in both the vertical and horizontal direction, and, thus, we decided to use the commercial suction cup for our adhesion module in the Limpet II. From our experiment, we also found out that the custom-made suction cups deteriorate quickly (3–4 days) and lose adhesion over time.

#### Design of the locomotion module

Locomotion is another important capability for inspection robots in offshore environments. We developed our locomotion module by using the EMM and rubber feet, as shown in Figure 3C. The locomotion is achieved by actuating the EMM at high frequencies, which results in vibrations. These vibrations are converted into a forward force that causes movement. Our locomotion technique is, thus, based on slip-stick locomotion.^21,112^ By changing the amplitude and frequency of vibration, or number of locomotion modules, we are able to achieve movement in different directions.

#### Design of the outer body (shell)

The shell acts as a protective housing for the Limpet II components. The shell was fabricated by using Ecoflex 00-50. We first developed a flat mold, which we used to make a 1 mm layer of Ecoflex 00-50. The soft layer was then bent into the right shape and used to cover the Limpet II components, with the sensing module exposed to the outer environment. The Ecoflex 00-50 is a soft material and, as such, can be manufactured at low cost and allows the Limpet II to be inherently complaint to its environment. In addition to that, the Ecoflex is lightweight (reducing the overall weight of the Limpet II), very strong (resistant to tearing), and very stretchy (capable of stretching multiple times of its original size without tearing and restores its original form without deforming).

### Fabrication

#### Fabrication of the PCBs

We designed the sensing and power driver PCBs by using Eagle PCB Design Software and fabricated them on double-sided Cu-FR4-Cu 0.1-mm boards. The sensing PCB and power driver PCB was fabricated by using an external company called Minnitron Ltd. (Kent, United Kingdom). We purchased all electronic components from RS Components, Digi-key Electronics, and Mouser Electronics. We soldered the components on the PCBs by using a reflow soldering process. In this process, we cut solder paste stencils from vinyl by using a Laser Cutter (Epilog Laser Fusion 32). We then stick the stencil on the PCB and apply solder paste on the stencil and PCB. We remove the stencil and manually place the components on their respective pads. Finally, we place the PCBs in an oven to reflow the solder.

#### Fabrication of the EMM

We fabricated the magnet holder, reel, and spring from acetate sheets by using kirigami. Kirigami involves cutting a pattern out of sheets and folding it into the desired three-dimensional (3D) configuration. The two-dimensional (2D) patterns for the coil reel, magnet holder, and spring are shown in Supplementary Figure S13. We use kirigami, as it allows our components to be low cost and highly manufacturable.^113^

The electromagnetic coil consists of a 280-turn 0.35 mm insulated copper wire. We used 12 mm permanent neodymium magnets for our EMMs. We produced the actuation coils for the EMM by using a custom-built coil-winding machine, which is shown in Supplementary Figure S14. We purchased the 0.35 mm insulated copper wire from RS components and the 12 mm permanent neodymium magnets from First4magnets. We used our custom-built coil-winding machine to wind the coils around the coil reel. The resulting reel with wound coils has an internal diameter of 14.5 mm, an outer diameter of 18.5 mm, and a height of 20 mm. The coil winding machine feeds the copper wire onto a rotating coil reel holder and we deposit superglue on the wire as it rotates so that the coil holds its shape.

We fabricated the top and bottom layers of the EMM from a 2 mm acrylic sheet. We used acetate for the spring of the EMM. Acetate provides advantages of low-cost, lightweight, and high manufacturability. Acetate is also a widely available material. We cut the patterns for the kirigami components (coil reel, magnet holder, spring) and the acrylic layers by using a laser cutter (Epilog Laser Fusion 32).

#### Fabrication of the adhesion module

We fabricated the adhesion module from an EMM, suction cup, vacuum pump, solenoid valve, and pressure sensor. We used 12 mm permanent magnets and a 280-turn 0.35 mm insulated copper wire coils for the EMM in the adhesion module. We drilled a hole in the suction cup and connected a plastic T-connector (3 × 1.8 × 0.6 cm) to it. We used gorilla glue to seal the connection between the T-connector and the hole in the suction cup. We used a 1.6 mm diameter silicon tubing to connect the T-connector to the pressure sensor and the valve. We used the same silicon tubing to connect the valve and pump. The EMM was then glued on top of the T-connector and suction cup.

#### Fabrication of the locomotion module

We fabricated the locomotion module from an EMM. Similar to the EMM fabrication, we used 12 mm permanent magnets and a 280-turn 0.35 mm insulated copper wire coils for the locomotion module. We added round black soft-plastic feet to the bottom layer of the EMM. The plastic feet have a height of 7 mm and a diameter of 12 mm. We used five soft-plastic feet on the locomotion module, where one foot was in the center of the module and the four others were each on one of the edges of the module (right, left, top, bottom).

## Experimental Design and Results

### Characterization of the EMM

In this section, we discuss the design of the experiments we used to demonstrate the basic capabilities of the EMM. We conducted three different experiments: frequency response analysis, evaluation of the spring constant, and measurement of the output force for different input actuation currents.

We conducted an additional experiment to analyze the effect of the coil wire thickness on the joule heating in the coils for different currents fed into the coil. In this experiment, we placed the coils with different thicknesses in a custom-designed white acrylic box containing a temperature sensor. The temperature sensor was connected to a computational circuit consisting of a microcontroller and bypass capacitors. We fed current into the coils and measured the temperature rise as a result of the input current. A picture of the experimental setup and results of this experiment are shown in Supplementary Figures S15 and S16, respectively. The results show that the amount of current drawn by the coils is proportional to the rate of temperature rise. We analyzed the temperature rise of the coils for a period of 150 s each. Each graph shows the rise in temperature over time for each of the coils at a different current level (1A, 2A, 3A, 4A). The results also show that the rate of temperature rise increases significantly as the wire thickness decreases.

#### Frequency response analysis

**FIG. 4.**
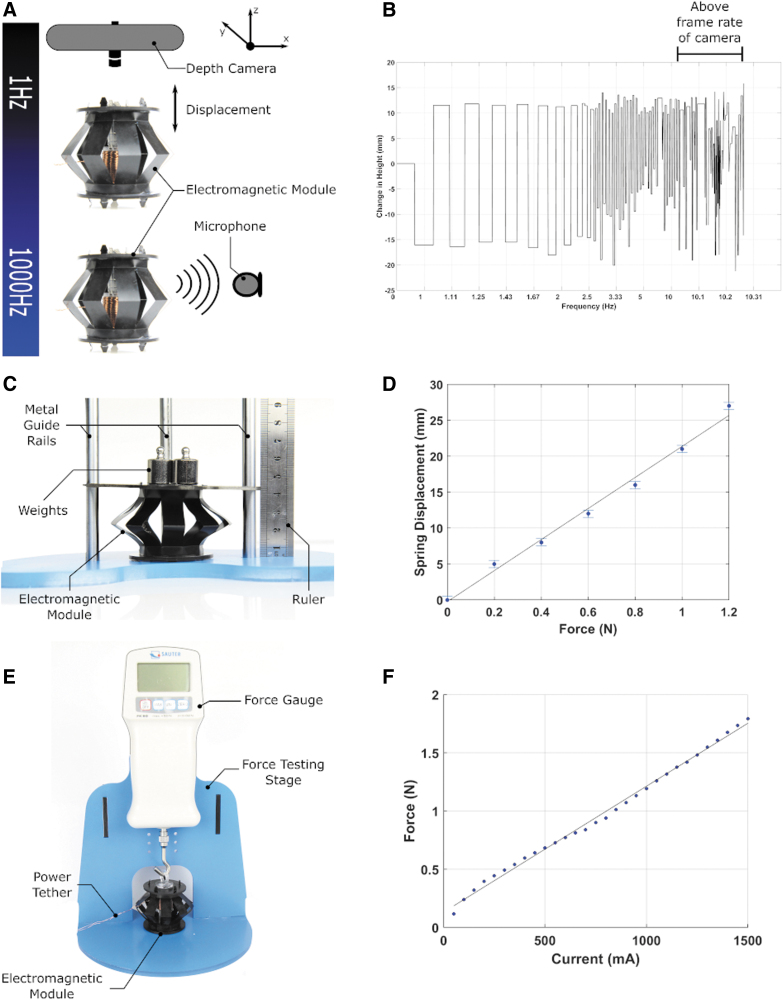
Characterization of the EMM. **(A)** Schematic of the experimental setup used to characterize the frequency response of the EMM. At low frequencies, the EMM demonstrates linear actuation capabilities and acts as a loudspeaker at higher frequencies. We used a depth camera to track the height of actuation at low frequencies, and a microphone to detect the sound output at higher frequencies. **(B)** Results of the frequency response analysis experiment showing the change in height of the EMM against the actuation frequency. **(C)** A labeled picture of the experimental setup used to measure the spring constant of the kirigami spring of the EMM. **(D)** Results of the spring constant evaluation experiment showing the extension of the spring against the force applied to the spring. **(E)** A labeled picture of the experimental setup used to measure the output electromagnetic force from the EMM for different current levels supplied to it. **(F)** Results of the experiment used to evaluate the output force from the EMM against the current supplied to the EMM. The experiment was repeated five times, and the results of all the five experiments are within the measurement error of the force gauge (±0.02 N). Color images are available online.

The EMM provides different capabilities over a wide frequency range. The primary function of the EMM is actuation at low frequencies, but it can also produce an audio output at higher frequencies. We designed an experiment in which we programmed the microcontroller to vary the coil frequency from 1 Hz to 15 KHz. We measured the height of actuation of the top part of the EMM by using a RealSense Camera mounted above the module. The RealSense Camera can detect the depth, and this function was used to track the movement of the top half of the EMM in the frequency response experiment. We also used an omni-directional sound sensor (−22 dB) to record the audio output produced by the EMM at high frequencies. The experimental setup is shown in Figure 4A. We also designed an experiment to test the pulse-width scheme on the EMM and its effect on the height of actuation by programming the microcontroller to provide a pulse-width modulating (PWM) signal to the coils of the EMM. We first sweep through duty cycles from 0% to 100% and then from 100% back to 0%. After that, we increment the duty cycles applied to the EMM in steps of 20% from 0% to 100%, and we measure the displacement at each step. We recorded the experiments by using an 18-mp Canon EOS 100D camera and an EF-S 18–55mm f/3.5–5.6 IS STM lens. The frequency response of the EMM can be seen in Supplementary Video S2. We also recorded the frequency response analysis experiment with a Sony PlayStation 3 eyetoy camera, which provides a higher frame rate than the RealSense camera. The video from the eyetoy camera is shown in Supplementary Video S3. A video of the PWM experiment can be found in Supplementary Video S4. We conducted these experiments only once to show the capabilities of the EMM over a range of different frequencies.

In this experiment, we demonstrate that the EMM can actuate at a low frequency and transition to sound output as the frequency increases to audible levels as shown in Supplementary Video S2. The video shows the EMM actuating at increasing frequencies and then transitioning from actuation to sound output at higher frequencies. The change in height of the EMM against actuation frequency at low actuation frequencies is shown in Figure 4B. As seen by the graph in Figure 4B, as the actuation frequency increases the period for the EMM to extend and contract fully decreases. As the frequency increases further, the change in height decreases as the EMM actuates at a very high speed. For frequencies higher than half of our camera frequency or frame rate, we get aliasing in the data as shown by the graph. Therefore, we can only trust data below the Nyquist frequency, which is half the frame rate of our camera. At very high frequencies, the camera cannot pick up the change in height properly as the EMM is actuating at a frequency higher than the frame rate of the camera. The sound spectrogram for the EMM at audible frequency levels is shown in Supplementary Figure S17. The spectrogram is based on the audio from seconds 24 to 42 in Supplementary Video S2. The audio is sampled at a rate of 44,100 KHz and divided into windows of 8820 samples with an overlap of 2205 samples between neighboring windows. Fast Fourier transform (FFT) is conducted on each window. The amplitude of the powers in the FFT is then scaled^114^ based on a Mel-Scale, which is a model of the sensitivity of the human ear to each frequency.^115^ The resulting FFTs are used to create a heat map of frequency versus time called a Mel-Spectrogram.

We also demonstrate how we can control the height of actuation by changing the PWM signal level. We expected that the use of PWM will change the actuation current to produce forces smaller than the maximum actuation force, and therefore, partial actuation. Our use of a PWM actuation signal produced the expected partial actuation behavior. The partial actuation behavior resulting from the PWM signal is demonstrated in Supplementary Video S4. The change in height of the EMM with respect to the PWM duty cycle is shown in Supplementary Figure S18. The change in height has a linear relationship with the PWM duty cycle.

#### Evaluating the spring constant of the EMM

The output force of the EMM is a combination of a spring force and an electromagnetic force. To evaluate the output force from the kirigami spring, it is important to study the elasticity of the kirigami spring by measuring its spring constant. The spring constant represents the amount of force required to compress or extend a spring. The relationship between the force applied to the spring and the displacement is given by:

F=kx

where *F* is the force applied, *k* is the spring constant, and *x* is the distance the spring is extended or compressed from its rest position. To evaluate the spring force, we designed an experimental setup to investigate the spring constant of the kirigami spring. Our experimental setup consists of the kirigami spring (without the coils and magnets), ruler, metal guide rails, and weights as shown in Figure 4C. In our experiment, we placed weights on the kirigami spring and measured the displacement of the spring. We increased the weight from 0 to 120 g in steps of 20 g. We used the metal guide rails to avoid any bending of the top acrylic part of the spring when we placed the weights on it. The experiment we conducted to identify the spring constant is shown in Supplementary Video S5. The graph of the force applied against the extension is shown in Figure 4D. The spring constant can be calculated by measuring the slope of the graph. In this case, the kirigami spring has a spring constant of 45.5 N/m. Since we took the measurements of displacement by using a ruler, the standard error is ±0.5 mm.

#### Determining the output force for different actuation currents

To determine the output force for different current levels supplied to the EMM, we designed a controllable experimental setup consisting of a force testing rig, force gauge, and a hook as shown in Figure 4C. We designed the setup so that the force gauge and the EMM are fixed in place in the force testing rig, with the force gauge aligned perpendicularly above the EMM. We connected the EMM and the force gauge together with a hook and a laser-cut eye loop. We programmed the EMM to contract at maximum force and, thus, it pulls against the force gauge hook and gives a force measurement on the force gauge. We supplied the EMM with different current levels and measured the maximum force achieved at each current level. We increased the current from 0 to 1500 mA in steps of 50 mA. When the EMM pulls against the force gauge, it does not change its shape. Thus, the effect of the spring force does not affect the results in this experiment. The electromagnetic force measured in this experiment is the force at zero displacement of the EMM. We repeated the experiment five times. Supplementary Video S6 shows one repeat of the experiment we conducted to measure the output force for different actuation currents. The output force from the EMM is a sum of the spring force from the kirigami spring and electromagnetic force from the interaction of the coils and permanent magnets. The total force generated by the EMM is given by:

$$F_{output}=F_{spring}+F_{electromagnet}$$

The electromagnet force, when the coil and magnets are fully separated, is given by:

$$F_{electromagnet}=\mu \frac{q_{coil}q_{pmag}}{4\pi {\left(d+r_{o}\right)}^{2}}$$

where μ is the permeability of free space, *q*_coil_ is the pole strength of the electromagnetic coil, *q*_pmag_ is the pole strength of the permanent magnets, d is the distance from rest position, and *r_o_* is the distance between the center of the coil and the center of magnets at rest position. *q*_coil_ is given by:

$$q_{coil}=\frac{NIA}{L}$$

where *N* is the number of turns of the electromagnetic coil, *I* is the current supplied to the coil, *A* is the cross-sectional area of the coil, and *L* is the length of the coil. In this experiment, the EMM was pulling against the force gauge with maximum force. The EMM did not change its height. Thus, the effect of the spring force does not affect the results of this experiment. For the electromagnetic force, the force measured is the force at zero displacement (d). The results of the output force for different actuation currents are shown in Figure 4F. There is a linear relationship between the current supplied and the actuation force. The results of all the five experiments are within the measurement error of the force gauge (±0.02 N). The maximum force achieved by the EMM in this experiment was 1.8 N, which suggests that a single module could lift objects with masses below ∼184 g when provided with an actuation current of 1500 mA.

### Characterization of the adhesion module

In this section, we discuss the design of the experiments we used to characterize the adhesion module of the EMM. We conducted two different experiments: strength of attachment to different surfaces and closed-loop control of the adhesion system. The first experiment evaluates the adhesion force of our adhesion module on surfaces with different roughness, and it gives a safety factor indication for the design of the adhesion module for different surfaces. The second experiment demonstrates the capability of our closed-loop controller implemented on the adhesion module.

#### Strength of attachment to different surfaces

To characterize the ability of our adhesion module to stick to different surfaces, we designed an experimental setup consisting of the adhesion module, surfaces with different average surface roughness, laser-cut acrylic weight rig, and weights. We measured the average surface roughness of five different surfaces by using the Dektak XTL stylus profiler: acrylic (5.318 nm), vinyl (172.276 nm), copper (365.814 nm), and two different types of steel (114.274 and 573.772 nm). The Dektak XTL provides a 2D roughness surface characterization by dragging a 2 μm stylus across the surface. The average surface roughness gives an indication of surface texture and overall profile height characteristics of a surface. For each surface, we repeated the surface roughness measurement five times, and we used the average of the five measurements. We conducted the experiment by attaching the adhesion module to each surface and testing the maximum vertical and horizontal loading capacity on the module. We attached the weight rig to the adhesion module and started adding weights to the rig in steps of 10 g until the adhesion module failed and detached from the surface.

**FIG. 5.**
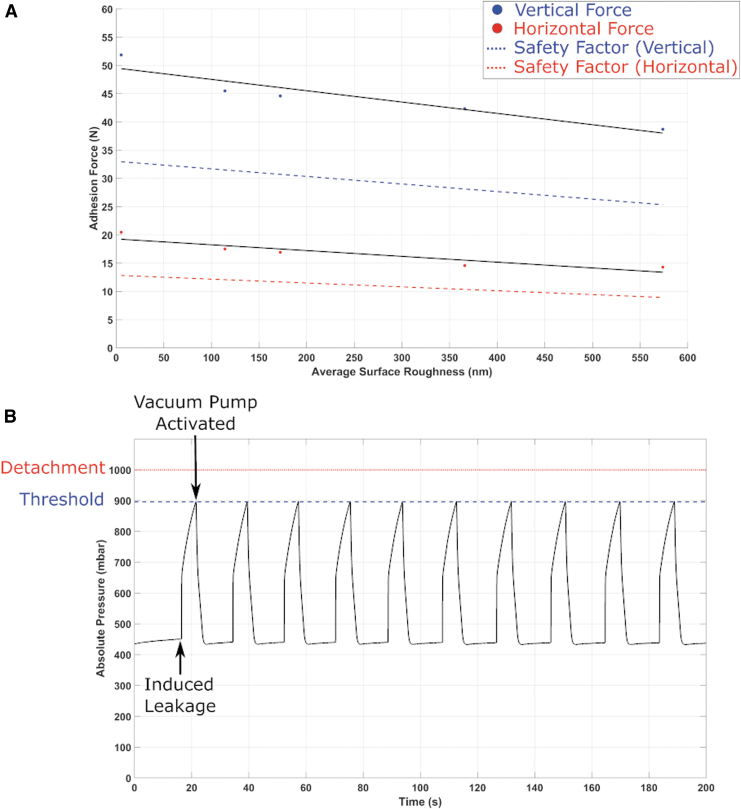
Characterization of the adhesion module. **(A)** Results of the adhesion force of the adhesion module on different surfaces. The graph shows the maximum vertical and horizontal adhesion force of the module for surfaces with different surface roughness, and a safety factor of 1.5 for the vertical and horizontal adhesion forces. **(B)** A graph demonstrating our closed-loop control of the adhesion module. We induce a leakage in the suction cup, which causes the pressure in the cavity between the suction cup and surface to rise. When the pressure reaches a certain threshold, which is lower than the pressure required to detach the suction cup, the valve will open and the pump will switch back on to restore the pressure in the cavity. Color images are available online.

In this experiment, we observe that the adhesion force of the adhesion module decreases linearly with average surface roughness. Figure 5A shows the relationship between the vertical and horizontal adhesion force of the adhesion module and the average surface roughness. We measured the average surface roughness of five different target surfaces as shown by the points on the graph. We then plotted the best fit line through the points. We added a safety factor of 1.5 for the vertical and horizontal adhesion force. Extensive literature is available about inner roughness of industrial pipes, as this is an important factor to estimate frictional losses in piping systems.^116,117^ Nevertheless, sources of external surface roughness are scarce. Steel is a material that is extensively used in industrial applications. The surface roughness of such materials depends heavily on its surface finish, which is a consequence of machining methods. For example, stainless steel surface roughness varies by grit size, depending on the finishing processes applied, which can range from 130 to 1800 nm.^118^

Taking into account the safety factor, the graph shows that on the surface with the highest measured surface roughness, the adhesion module can handle up to 26 N in the vertical direction and up to 9 N in the horizontal direction. These measurements indicate that the adhesion module can handle an average maximum weight of 920 g in the horizontal direction if placed on a rough metallic surface. We used this measurement as a guide for the maximum allowed weight of the final Limpet II system, if it is to be used for climbing rough surfaces in offshore environments.

#### Closed-loop control of the adhesion system

We designed a closed-loop controller for our adhesion system. The closed-loop controller monitors the pressure in the cavity between the suction cup and the surface. When the pressure reaches a certain threshold, which is lower than the pressure required to detach the suction cup (atmospheric pressure), the controller will open the valve and turn on the vacuum pump to restore the pressure in the cavity. The controller ensures that if any leakage occurs in the suction cup, the adhesion module will not detach from the surface. To test the efficiency of the controller, we designed an experimental setup where we induce a leakage in the suction cup by opening the solenoid valve to the atmosphere and then monitoring the effect of the closed-loop controller on restoring the suction cup pressure.

In this experiment, we first attach the adhesion module to the surface by pumping the air out of the cavity between the suction cup and the surface. At this point, the pressure of the suction cup is measured to be 435 mbar. The suction sup detaches from the surface when the pressure in the cavity reaches atmospheric pressure (1014 mbar). We developed a closed-loop controller that monitors the pressure in the cavity, and if the pressure ever reaches 895 mbar, the controller will open the pump to restore the adhesion force to normal. We decided to activate the pump at a much higher pressure to the required pressure (895 mbar as compared with 435 mbar) to reduce the energy consumption required to maintain the closed-loop adhesion, and as such extending the battery life. In this experiment, we induced a leakage in the suction cup after attachment, by opening the valve to the atmosphere, and monitored the pressure as it rose toward atmospheric pressure. When the pressure reached 895 mbar, the pressure was restored back to 435 mbar as shown in Figure 5B. We repeated this procedure multiple times, and the closed-loop controller was always successful in restoring the adhesion of the module and preventing detachment of the suction cup.

### Characterization of the locomotion module

In this section, we discuss the design of the experiments that we used to characterize the locomotion module of the Limpet II. This section is divided into two parts. In the first part, we designed an experiment to show the modularity aspect of our system, and how we can achieve simpler controllers and more complex systems by stacking basic modules (EMMs) together. In the second part, we conducted an experiment to track the movement of our final locomotion system as it moves in a predefined geometry in its environment. We also conducted an experiment to track the displacement of the top part of the final locomotion system as it moves in a straight line along one axis.

#### Effect of stacking the locomotion modules

The locomotion using this module (EMM and rubber feet) can be achieved in multiple different ways depending on the number of modules used and the mechanical design of the system. The mechanical design of the system will affect the center of mass of the system. In this section, we designed an experiment to investigate the effect of stacking or combining basic functional modules (locomotion modules) to create a system that is more complex and has greater capability than the sum of its individual parts. We use three types of locomotion systems: a single EMM, two EMMs, and four EMMs as shown in Figure 3C–E. In this experiment, we have an environment with four targets in each of the four cardinal directions. The aim of this experiment is to show how we can stack or combine locomotion modules to produce a system that can achieve greater capability with simpler control. In the first part of the experiment, we used a single locomotion module and show the effect of weight biasing on the ability of module to reach the targets. We used a 20 g weight to achieve the change in directions by using the single locomotion module. We used an actuation frequency of 4 Hz, and we supplied the module with an actuation current of 300 mA. In the second part of this experiment, we demonstrated the capability of a system consisting of two combined locomotion modules and showed the effect of both amplitude biasing and weight biasing on the performance of this system. For weight biasing, we change the direction of movement of the system by using a 20 g weight; whereas for amplitude biasing, we actuate each of the locomotion modules in a different way from the other. The two locomotion modules are attached together by using a 2 mm acrylic piece. For this part of the experiment, one module was contracted fully whereas the other module was actuating at a frequency of 6.7 Hz. We supplied an actuation current of 750 mA to both modules. Finally, we designed a system with four different modules, used amplitude biasing on the system to try to reach the four targets, and showed the effect of this system on our controller and system design.

**FIG. 6.**
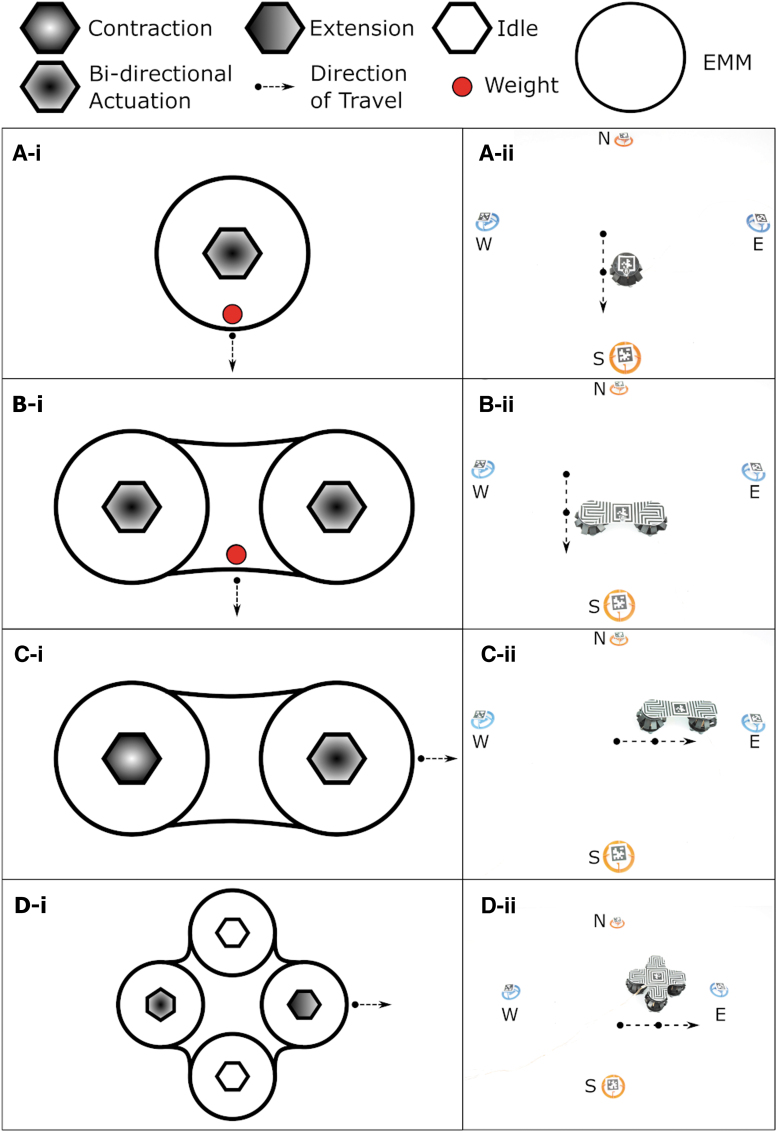
Modularity and stacking of the locomotion modules. **(A-i)** Schematic and **(A-ii)** Image of a single locomotion module biased to move to a target in the South direction by adding a weight on one side of the locomotion module to bias it to move in that direction. **(B-i)** Schematic and **(B-ii)** Image of a locomotion system made up of two EMMs biased to move to a target in the South direction by adding a weight on top of it. **(C-i)** Schematic and **(C-ii)** Image of a locomotion system made up of two EMMs biased to move to a target in the East direction by actuating the two EMMs in different manners to each other. **(D-i)** Schematic and **(D-ii)** Image of a locomotion system made up of four EMMs biased to move to a target in the East direction by actuating one EMM in both directions, contracting the EMM opposite to the bi-directional EMM, and keeping the other two EMMs in idle state. Color images are available online.

In this experiment, we observed that using one locomotion module to reach four targets, one in each cardinal direction, requires a change in the mechanical structure of the module. We can bias the direction of movement by placing a weight at the edge of the EMM facing the desired direction of movement. We use a 20 g weight for this experiment, and we repeat the experiment five times for each of the four cardinal directions. We have to manually change the location of the weight on the top acrylic layer of the module to change the direction of motion. One repetition of the experiment is shown in Figure 6A. The weight tilts the EMM and shifts its center of mass, allowing it to move to the desired direction. Thus, to use a single EMM as the final locomotion system, we would have to design a mechanical system that can shift the center of mass of the EMM toward the required direction, and we would have to develop a technique to allow the EMM to alter that mechanical system autonomously to achieve successful movement. This approach of using a single EMM would make the mechanical design and the controller of the Limpet II very complex. The average speed of the single module in this experiment is 35.4 mm/s. The single locomotion module deviates from its path by 20 mm in the North direction, 17 mm in the South direction, 19 mm in the East direction, and 22 mm in the West direction. Supplementary Video S7 shows the single locomotion module reaching the four targets by biasing the direction using an external weight. When we stack two EMM modules into one locomotion system, the system is capable of more complex motions with a simpler controller and design. The system can be biased in two ways. The center of mass of the system can be shifted by using an external weight or using a combination of the two EMMs. The first method involves adding a 20 g weight on either side of the space between the two EMMs. The weight tilts the system and results in a shift in the center of mass, causing the locomotion system to move in the north or south directions. Movement of the two EMMs in the south direction by using mass biasing is shown in Figure 6B. The second method involves programming the EMMs to have different behaviors, which will also result in a shift of the center of mass of the system. In our case, we program the EMM that is on the side closer to the target to actuate continuously in both directions, whereas the EMM furthers away from the target to be in continuous contraction at maximum force as shown in Figure 6C. We repeated the experiments five times for each of the different directions. The average speed achieved by the system when biased by the weight is 70.7 mm/s. The average speed achieved by biasing the system using the combination of the EMMs is 31.4 mm/s. The two-module locomotion system deviates from its path by 11 mm in the North direction (weight bias), 22 mm in the South direction (weight bias), 15 mm in the East direction (amplitude bias), and 17 mm in the West direction (amplitude bias). The results of the locomotion system composed of two EMMs are shown in Supplementary Video S8. Based on the results of these two experiments, we decided to develop a locomotion system by using four EMMs. We can use this locomotion system to achieve motion in all cardinal directions by using only the EMMs. An example of the movement in the west direction is shown in Figure 6D. We use the EMMs to shift the center of mass of the locomotion system and achieve movement in the required direction. Thus, this system simplifies our mechanical design and the controller required. We repeated the experiments five times for each of the different directions. The average speed achieved by this system is 37.1 mm/s. The four-module locomotion system deviates from its path by 4 mm in the North direction, 15 mm in the South direction, 14 mm in the East direction, and 17 mm in the West direction. All the targets are reached successfully and simply by the locomotion system without using any external weight bias, and the four-module locomotion system achieves the least deviation in its path compared with the other locomotion systems. The results of the locomotion system composed of four EMMs are shown in Supplementary Video S9. The speed of the different systems is not an important comparison parameter, as we can control the speed of the system by changing the input current and ctuation frequency of the different modules. The locomotion modules in the Limpet II system can also be programmed to achieve diagonal movement as shown by Supplementary Video S18.

#### Evaluating the locomotion

We observe that stacking functional blocks results in systems that are increasingly capable of a diverse range of complex motions and behaviors. We decided to use the locomotion system composed of four EMMs and explore it further. We conducted two different experiments to evaluate the locomotion of the system. We recorded both experiments by using an 18-mp Canon EOS 100D camera and an EF-S 18–55mm f/3.5–5.6 IS STM lens.

**FIG. 7.**
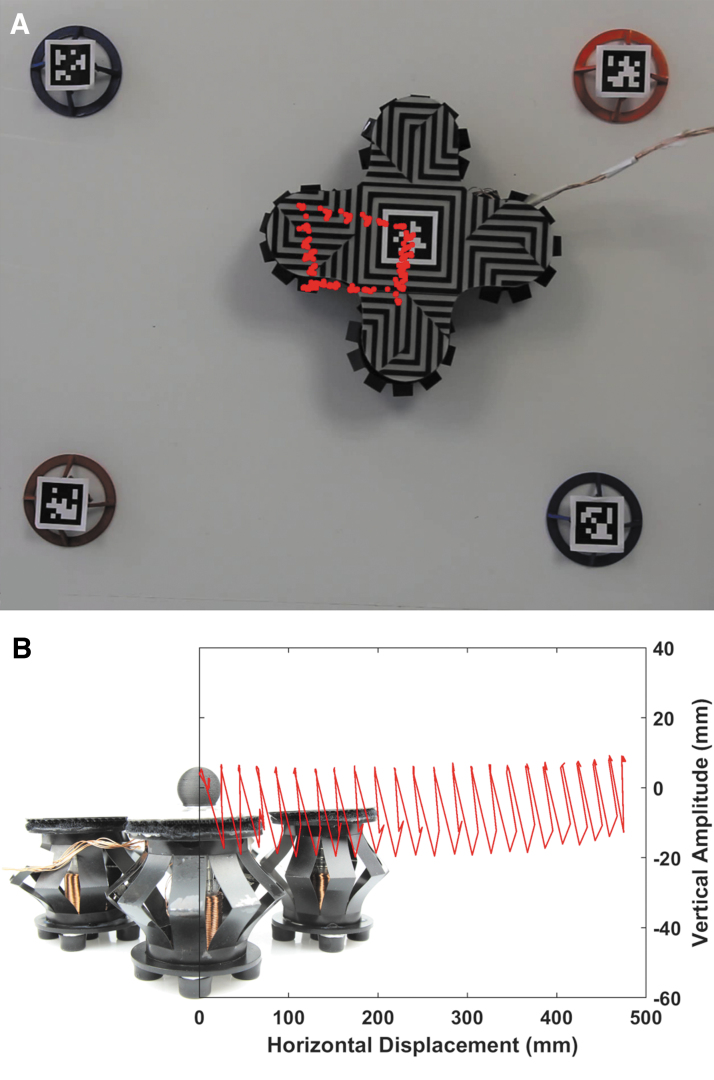
Characterization of the locomotion system with the four locomotion modules. **(A)** The path of the locomotion system after being programmed to travel in a rectangular path and the position of the system is tracked by using AprilTags. **(B)** Tracking the vertical displacement of the locomotion system as it moves horizontally in a *straight line*. Color images are available online.

In the first experiment, we programmed the system to travel in a rectangular path and we tracked the position of the system by using AprilTags.^119^ Each side of the rectangular path is achieved by actuating a different combination of locomotion modules at different frequencies. The locomotion system is programmed to move in a rectangular fashion and can achieve so with a slight deviation in one of the sides as shown in Figure 7A and Supplementary Video S10. We supplied a current of 2A to the system in this experiment.

In the second experiment, we track the change in height of the top part of the locomotion system as it moves in a straight line along one axis as shown in Supplementary Video S11. We track the displacement and change in height by using a 3D-printed black sphere that we attached to the top of the locomotion system. We developed a custom-written script that can track the black sphere against the white background and record the change in position of the sphere both vertically and horizontally. The change in height versus horizontal displacement is shown in Figure 7B.

### Demonstrations

In addition to the experiments that show the characterization of the different modules included in the Limpet II, we performed additional demonstrations to show how the modules can be configured to allow the Limpet II to perform useful behaviors in its environment.

#### Untethered sensory control of the Limpet II

In this experiment, we use the different sensors on the Limpet II to control its locomotion and adhesion capabilities. We use the sound, distance, light, and inertial measurement unit (IMU) sensors to show an example of how the Limpet II can respond to a sensory input in its environment. These demonstrations show how the Limpet II can use this capability and the sensing measurements to map its environment and act on changes in that environment. These demonstrations also show how the Limpet II can perform structural monitoring and fault detection (e.g., finding leaks in pipes), where the Limpet II needs to respond to a change in the monitored value (measurand) to identify the location of the fault. Such capabilities (environmental monitoring, structural monitoring, and fault detection) are important aspects for inspecting and monitoring offshore platforms. Supplementary Video S12 shows the Limpet II responding to a light stimulus, where a change in the ambient light color will lead to movement in different directions. In this demonstration, we use the light sensor on the Limpet II, which is capable of measuring ambient light intensity, and power density of red, green, and blue light. Supplementary Video S13 shows how the Limpet II can change its movement direction or enable and disable its adhesion mechanism based on a change in frequency of a sound input signal that is measured by the sound sensor on the Limpet II. Supplementary Video S14 shows how the Limpet II can respond to the presence of objects at different proximity from the Limpet II by using the on-board distance sensor. In Supplementary Video S15, we poke the Limpet II, which will cause it to start repositioning itself away and then adhering in place at the new position. We use the IMU sensor for this demonstration, where poking the Limpet II will result in a small change in the acceleration and gyro data. This small change is picked up by the Limpet II, which responds to this change by repositioning itself in its environment.

#### Sound-based intercommunication of limpets

One of the capabilities of the EMM is generation of sound output. This capability can be used by the Limpet II for communication with neighboring Limpets. In this demonstration, we demonstrate how the sound capabilities of the EMM in the Limpet II can be used for communication with other neighboring Limpets. One Limpet II generates a sound signal (distress signal), and the neighboring Limpet II around it will start moving away from that Limpet II. The result of this demonstration is shown in Supplementary Video S16. This capability can be used in a situation where other communication systems (e.g., WiFi and LoRa) are down, and the Limpet II needs to communicate to its neighbors. The idea behind this capability is that the Limpet II can send out command signals to its neighbors, which can be, for example, to start inspecting and mapping the structure for faults.

#### Climbing

To highlight the modularity aspect of the Limpet II system and to show its capability in unstructured offshore environments, we reconfigured the modules of the Limpet II to enable it to climb an inclined surface (with an average surface roughness of 6 nm). A side view and a top view of the Limpet II climbing the surface is shown in Supplementary Video S17. In this experiment, we reconfigured our electromagnetic and adhesion modules, as shown in Supplementary Figure S19. The Limpet II configuration includes two vertical adhesion modules, and two horizontal EMMs connected between the adhesion modules. We programmed the Limpet II to follow a sequence of steps as follows:

- Top adhesion module adheres to the surface by activating its pump, closing its valve, and extending itself to push the suction cup on the surface.
- Bottom adhesion module contracts to lift its suction cup off the surface.
- Horizontal EMMs contract, which brings the bottom adhesion module closer to the top one.
- Bottom adhesion module adheres to the surface by activating its pump, closing its valve, and extending itself to push the suction cup on the surface.
- Top adhesion module detaches from the surface by opening its valve to the atmosphere and contracting to lift its suction sup off the surface.
- Horizontal EMMs extend, which pushes the top adhesion module further away from the bottom one.
- Top adhesion module adheres to the surface by activating its pump, closing its valve, and extending itself to push the suction cup on the surface.
- The motion is then repeated multiple times to achieve the vertical locomotion on the surface.

## Discussion

In this work, we present an untethered hybrid robot that integrates a sensing module, power driver module, and adhesion and locomotion modules based on EMMs. The robot is designed in a modular fashion, equipped with nine exteroceptive sensing modalities, and feature capabilities of simple and untethered control, adhering to different structures and materials (acrylic, vinyl, copper, steel), walking on horizontal surfaces, climbing inclined surfaces, and sensing its environment.

The Limpet II is a useful system for carrying out inspection tasks offshore, as it is capable of navigating on surfaces at different angles. Current climbing robots focus on the climbing capabilities and have limited or no sensing abilities on-board. Also, the existing systems mostly use rigid actuators, which does not allow the robot to easily conform to its environment and adds complexity in the task planning phase. Soft climbing robots that have been reported^63^ are not yet fully untethered systems, which limits their capability to carry out useful tasks in such extreme environments. The Limpet II uses hybrid actuators, which has the advantages of rigid components and can still partially conform to its environment. The Limpet II is also developed as a fully, untethered system, which removes the limitations associated with power and communication tethers in performing tasks and allows for autonomous deployment of the system. The Limpet II is equipped with nine sensing modalities, which allows it to sense its environment and monitor the conditions of the different offshore structures. The Limpet II is developed in a modular fashion, which makes it easier to adapt the system to new tasks, to repair the system, and to replace and reconfigure the modules.

One major drawback of the locomotion technique of the Limpet II is that there is no real form of on-board odometry, which makes moving accurately and precisely for a long duration of time or for large distances a challenging task. However, we can achieve odometry by using a combination of the AprilTags surrounding the system and an off-board communication system (e.g., IR transceiver), to accurately estimate the change in position and achieve more precise movement over long distances. Another limitation with the current system is the high power consumption of EMMs when holding force. We try to minimize this power consumption by reducing the time in which EMMs hold their force. For adhesion, the module is provided with a single power impulse to help with the adhesion process and then the pump kicks in and the EMM is switched off. For the locomotion, the EMM is provided with power impulses in both directions, and we minimize the time between pulses to reduce the holding force period of the module. The Limpet II system can achieve vertical and inverted climbing by reconfiguring the modules and adding extra horizontal modules to provide a higher pulling force.

We can easily alter the speed of the locomotion system, as the speed is a function of the current supplied, frequency of actuation of the EMMs, and the weight of the system. However, there are some limitations to increasing the speed, as faster actuation consumes higher power and affects the battery lifetime. Power consumption during deployment life is a very important consideration for the Limpet II system to be used in offshore environments. The speed achieved in vertical climbing is smaller than that achieved in horizontal motion, as the adhesion and locomotion forces are used to overcome the gravity force of the robot during climbing. The adhesion capability of the Limpet II relies on materials and surface textures, and it works best on flat surfaces. The adhesion can be used on rough and uneven surfaces, but it will achieve shorter periods of adhesion. The roughest surface that the Limpet II can manage to climb can be identified by using the surface roughness figure (Fig. 5A); the limpet II can climb any surface where its weight is less than the adhesion force for that specific surface roughness. The Limpet II system presented in this work has a short battery life. The battery life can be increased by using larger batteries, but those batteries have larger weight and will affect the adhesion force and the climbing speed of the system.

## Conclusion

Existing climbing robots mostly use rigid actuators, and robots that use soft actuators are not fully untethered yet. Current climbing robots are also not built in a modular fashion, which makes it harder to adapt the system to new tasks, to repair the system, and to replace and reconfigure the modules. In this work, we present an untethered hybrid soft/hard robot—the Limpet II—that is designed in a modular fashion. The modules of the Limpet II can be reconfigured to achieve different tasks, giving it the ability to climb inclined surfaces, adhering to different structures, and sensing its environment. The Limpet II uses a hybrid EMM as its core unit to allow adhesion and locomotion capabilities. The adhesion capability is based on negative pressure adhesion utilizing suction cups. The locomotion capability is based on slip-stick locomotion. The Limpet II also has a sensor payload with nine different sensing modalities, which can be used to inspect and monitor offshore structures and the conditions surrounding them. To demonstrate its potential for inspection of offshore platforms, we show that the Limpet II is capable of responding to different sensory inputs, repositioning itself within its environment, adhering to structures made of different materials, and climbing inclined surfaces.

## Supplementary Material

Supplemental data

Supplemental data

Supplemental data

Supplemental data

Supplemental data

Supplemental data

Supplemental data

Supplemental data

Supplemental data

Supplemental data

Supplemental data

Supplemental data

Supplemental data

Supplemental data
