# Supplementary material for: Limpet II: A Modular, Untethered Soft Robot
Source: Soft Robot. 2021 Jun 16;8(3):319–39. doi: 10.1089/soro.2019.0161 (PMC8236390; doi:10.1089/soro.2019.0161)
Supplement: Supplemental data [file Supp_Videos.docx]

# Video Captions and Links

**Video S1: Overview of the Limpet II System.**

<https://www.youtube.com/watch?v=i2o2LDF2FoA&feature=youtu.be>

**Video S2: Frequency Response Analysis.** A video showing the effect of varying the control frequencies on the EMM. The EMM actuates at low control frequencies, and as the frequency increases, the EMM transitions to producing sound.

<https://www.youtube.com/watch?v=CZ0GgVW7At0&feature=youtu.be>

**Video S3: Frequency Response Analysis at a Higher Frame Rate.** A video showing the frequency response of the EMM using a camera with a higher frame rate.

<https://www.youtube.com/watch?v=CZ0GgVW7At0&feature=youtu.be>

**Video S4: Pulse-Width Modulation of the Electromagnetic Module.** A labelled video showing a pulse-width-modulated (PWM) signal applied to the EMM to observe the effect of changing the duty cycle on the actuation height. The EMM sweeps through duty cycles from 0-100% and then from 100-0%. The EMM then steps between set duty cycles of 20%, 40%, 60%, 80% and 100%.

<https://www.youtube.com/watch?v=JbNIjt4uQ5Q&feature=youtu.be>

**Video S5: Evaluation of the Spring Constant of the Electromagnetic Module.** A video showing the experiment conducted to evaluate the spring constant of the EMM, where weights are added on the spring and the change in height measured.

<https://www.youtube.com/watch?v=vT4hkd5p6h8>

**Video S6: Determining the Output Force for Different Actuation Currents.** A labelled video showing the setup for the quantification of force output from the EMM. The video shows the output force for different current levels supplied to the EMM.

<https://www.youtube.com/watch?v=rlc4Ros7erQ>

**Video S7: Single Locomotion Module.** A video showing a single locomotion module reaching targets in the four cardinal directions in an environment. We bias the direction of movement of the module by changing the position of the weight on top of it.

<https://www.youtube.com/watch?v=UV21JiEQnmQ>

**Video S8: Locomotion System with Two Locomotion Modules.** A video showing a locomotion system composed of two locomotion modules reaching targets in the four cardinal directions in an environment. We bias the direction of movement of the module by changing the position of the weight on top of it or changing the actuation pattern of the two modules with respect to each other.

<https://www.youtube.com/watch?v=kvH2mKHeORQ>

**Video S9: Locomotion System with Four Locomotion Modules.** A video showing a locomotion system composed of four locomotion modules reaching targets in the four cardinal directions in an environment. We bias the direction of movement of the module by changing the actuation pattern of the locomotion modules with respect to each other.

<https://www.youtube.com/watch?v=yD9VjeU7m0I>

**Video S10: Locomotion System Following a Rectangular Shaped Path.** A video showing the tracking of the locomotion system as it moves in a pre-programmed rectangular shape.

<https://www.youtube.com/watch?v=d4DrnmgM88E>

**Video S11: Tracking the Height of Displacement of the Locomotion System.** A video showing the locomotion system moving horizontally on a surface in a straight line. The black sphere on the locomotion system is used to track the height of movement of the locomotion system as it moves along the surface.

<https://www.youtube.com/watch?v=liaZ1zQOaTo&feature=youtu.be>

**Video S12: Untethered Control of the Limpet II Using Light Sensor.** A video showing the Limpet II responding to a change in the LED colour by changing direction of movement.

<https://www.youtube.com/watch?v=3eP2ip_yieM>

**Video S13: Untethered Control of the Limpet II Using Sound Sensor.** A video showing the Limpet II responding to a change in sound frequency by changing direction of movement.

<https://youtu.be/enTPQxcJqto>

**Video S14: Untethered Control of the Limpet II Using Distance Sensor.** A video showing the Limpet II responding to the presence of an object in close proximity by repositioning itself.

<https://www.youtube.com/watch?v=zwd-71A086s>

**Video S15: Untethered Control of the Limpet II Using IMU Sensor.** A video showing the Limpet II repositioning itself when it is poked or touched.

<https://www.youtube.com/watch?v=81w0ljYadIQ>

**Video S16: Sound-Based Inter-Communication of Limpets.** A video showing one Limpet II generating a sound signal (distress signal), and the neighbouring Limpet OO repositioning itself away from the first Limpet II.

<https://www.youtube.com/watch?v=tl0-oEvpvMM>

**Video S17: Climbing an Inclined Surface.** A video showing the top and side views of the Limpet II climbing a surface.

<https://www.youtube.com/watch?v=ujTccxPR4wA&feature=youtu.be>

**Video S18: Diagonal Movement of the Limpet II System.**

<https://www.youtube.com/watch?v=stcTeIlWMVg&feature=youtu.be>
