# Supplementary material for: Limpet II: A Modular, Untethered Soft Robot
Source: Soft Robot. 2021 Jun 16;8(3):319–39. doi: 10.1089/soro.2019.0161 (PMC8236390; doi:10.1089/soro.2019.0161)
Supplement: Supplemental data [file Supp_Data.pdf]

Supplementary Data

Supplementary Materials and Methods

The Limpet II

The Limpet II is being developed as part of the Offshore Robotics for Certification of Assets (ORCA Hub) in the United Kingdom. The ORCA Hub is a collaboration between five universities (University of Edinburgh, Heriot-Watt University, University of Liverpool, Imperial College London, and University of Oxford) and top industrial companies (Total, Chevron, British Petroleum, Tharsus, and many others). The ORCA Hub aims at assisting industries in moving toward an offshore platform that is operated, inspected, and certified by autonomous robots.

The Limpet version 1 was designed to be low cost and highly manufacturable, and thus it can be deployed in huge collectives for monitoring the offshore platforms. Version 1 of the Limpet is an integrated Printed Circuit Board (PCB) with nine sensing modalities. We integrated it with Robot Operating System, to allow it to interact with other robots on the offshore platform. We demonstrated how the Limpet version 1 can be used to achieve real-time condition monitoring for offshore structures, by combining remote sensing with signal-processing techniques, where the Limpet detects a fault in the operation of an offshore asset by running an on-board classifier.[S1]

Sensing modalities and programming

The sound sensor on the sensing module has an analog output, whereas all the other sensors are digital sensors controlled through the I^2C bus. The sound sensor has an analog output and is connected to an analog-to-digital converter on the microcontroller. Analog sensors produce a continuous analog output signal that is proportional to the measurand. Digital sensors, on the other hand, convert the measured signal into a digital output signal inside the sensor itself, and they then transmit the digital signal. Digital sensors are usually preferred over analog sensors, as they are less susceptible to noise (undesirable variation in voltage). Small variations in the voltage of an analog signal can result in significant errors during the processing stage. Thus, digital sensors have a higher accuracy and throughput.

The sensing module and power driver module have an on-board programming port (JTAG). We program the Limpet II via a SEGGER J-Link programmer together with a JTAG Adapter (Olimex ARM-JTAG-20-10) by using a serial wire debug interface. We program the Limpet II in C/C++ by using Atmel Studio 7, and the main code includes online libraries and custom-written sensor libraries. We used the standard Universal Asynchronous Receiver-Transmitter (UART) protocol for communication. The UART protocol uses a high idle line, which is pulled low at the start of a message.

Design of the sensing and power driver modules

The sensing module consists of a single two-layer PCB incorporating a low-power microcontroller (ATSAMD21-G18A), a 32.768 KHz crystal oscillator (CM7V-T1A), RGB LED (LTST-N683EGBW), charging IC (MCP73812), programming port [JTAG] (Molex 532610571), and a communication connector as shown in Figure S4. The PCB includes several exteroceptive sensors, which are: temperature and humidity sensor (Si7006), inertial measurement unit (Accelerometer and Gyroscope) sensor (LSM-6DS3), optical sensor (VEML6040), sound sensor (SPU0414HR5H-SB), 3-Axis magnetic sensor (MLX90393), pressure sensor (BMP280), and distance sensor (VL53L0). The communication connector on the sensing module is connected to the UART of the microcontroller. Therefore, we can use this connector to connect the sensing module to multiple different communication systems. We connect the communication connector to the power driver module. The microcontroller on the sensing module is responsible for controlling the sensing capabilities, and sending control messages to the power driver module based on the sensory input.

The power driver module consists of a single two-layer PCB incorporating a low-power microcontroller (ATSAMD21-G18A), H-bridge drivers (DRV8871 and DRV8837), solenoid valve, air pump, barbed absolute pressure sensor (HSCMANN 015PA2A3), 3.5 V voltage regulator (LP2985IM5), 5 V voltage regulator (MC78L05ACDR2G), 32.768 KHz crystal oscillator (CM7V-T1A), infrared (IR) transceiver (TFBS4711), connection to sensing module, battery connector, programming port, and a communication connector. We use the microcontroller to control the frequency of the actuation of the electromagnetic modules (EMMs). We control the direction of the actuation of the EMMs by the H-bridge driver using the microcontroller. The air pump and solenoid valve are also controlled by the microcontroller through an H-bridge. The microcontroller on the power driver module is responsible for controlling the adhesion and locomotion capabilities. The pressure sensor is used as a feedback component for the closed-loop controller of the adhesion system. The sensing module sends all the on-board sensor data to the power driver PCB, which then sends it to the computer through the IR transceiver or any other communication system (serial, WiFi, LoRa, optical) connected to the communication connector.

Design of the communications for the Limpet II

To use WiFi for transmission of data from the Limpet II to a node (e.g., PC), we use an ESP8266, which is a low-cost System-on-a-Chip WiFi module with integrated TCP/IP protocol stack that is capable of giving any microcontroller access to the WiFi network. We use a software development kit, known as NodeMCU, to program the chip directly. NodeMCU is a Lua-based firmware, where Lua is lightweight multiparadigm programming language that is used for embedded applications. To send and receive the messages between the ESP8266 and the PC, we use Eclipse Mosquitto,[S2] which implements a messaging protocol known as Message Queuing Telemetry Transport (MQTT). MQTT is a lightweight publish/subscribe messaging protocol used for remote communication as shown in Figure S1. In this case, the

Limpet II is the publisher and the PC subscribes to the MQTT topic to receive the Limpet II data. The received data can then be plotted in a real-time basis, or they can be saved on the PC and processed later.

We transmit the data from the Limpet II serially by using SparkFun's FTDI Basic Breakout-3.3 V, which is a serial to the USB convertor. The communication between the Limpet II and the FTDI Basic Breakout occurs over UART. The transmitting UART (Limpet II) converts parallel data from the microcontroller into serial format, transmits it into serial format to the receiving UART (FTDI), which converts the serial data back into parallel form for the PC. The UART transmits the data asynchronously, which means there is no clock synchronization between sending data from the transmitting device and receiving data by the receiver. In UART, instead of the clock signal, the transmitting device adds a start bit and a stop bit to the data being sent, which defines the beginning and end of the data packet for the receiving UART. A schematic of the serial communication is shown in Figure S2.

LoRaWAN is a new long-range wide area network technology that allows for long-range communication with low power consumption, but with a limited bandwidth.[S3] There is a tradeoff between the spreading factor (SF) and communication range in LoRa. The higher the SF, which means a slower transmission, the longer the communication range. Depending on the SF used (7 to 12), LoRaWAN data rate ranges from 0.3 to 27 kb/s.[S4] We used the LoPy, which is a MicroPython-enabled Wi-Fi, Bluetooth, and LoRa development board, to gain access to the LoRa network. In LoRa communication, devices or nodes send small data packets to a gateway, which can be several kilometers away from the node, using the LoRaWAN wireless protocol. The gateway then uses traditional communication methods (Ethernet, WiFi, mobile network) to forward the packets to a network server, which validates the packets and forwards them to an application server. Since the Limpet II is designed to be used in offshore energy platforms, where data security is important and internet connectivity is low, we have developed a local network for the LoRaWAN communication for the Limpets. The local LoRaWAN network can be used in any environment and allows the data packets to be secure. The local network developed uses the LoRa Server project provided by CableLabs.[S5] The architecture of the LoRa network is shown in Figure S3. The Limpet II sends the sensor data through the UART to the LoPy nodes, which send these data packets to the LoRa gateway by using the LoRaWAN wireless protocol. The gateway runs a packet-forwarder software, which is responsible for forwarding the incoming packets to a network server by using a User Datagram Protocol (UDP). The network server and the LoRa gateway are given IP addresses through a local Domain Name System server. The LoRa Gateway Bridge, which runs on the local machine or PC that runs the network server, is responsible for transforming the packet-forwarder UDP Protocol into an MQTT Protocol. We used Eclipse Mosquitto as the MQTT Broker. The MQTT messages are forwarded to the LoRa Server, which is the LoRaWAN network server component running on the local machine. The LoRa Server manages the state of the network, where it handles the join requests by the devices and has knowledge of which devices are active on the network. The LoRa App Server component implements a LoRaWAN application-server compatible with the LoRa Server component. It provides a web interface and APIs for management of users, organizations, applications, gateways, and devices. The data payload sent by the devices can then be published from the LoRa App Server to an application by using MQTT protocol.

Optical communication has a low bandwidth, and, thus, the processing and analysis need to be done before transmission of the data. For optical communication, we use the on-board RGB LED for transmission of data. We transmit the data by pulse-width modulating (PWM) the LED to different levels, where each level corresponds to a number from 0 to 9. We pulse-width modulate the RGB LED from 0 to 255 in steps of 25, which allows for 10 different intensity levels corresponding to each of the numbers (0 to 9). We use the different LED colors to correspond to different measurements in a single experiment. The optical communication protocol transmits a single digit at a time. Therefore, we programmed the Limpet II to divide any measurements into single digits and transmitted the digits one after the other. The intensity of the transmission (PWM level) and the LED color (measurement type) allows reconstruction of the transmitted data from the RGB LED. We use the optical sensor on the Limpet II as a receiver, where it detects the light intensity (indication of the transmitted number) and color power density (indication of measurement type), to interpret the data transmitted by using the RGB LED. The data transfer speed achieved in our optical communication protocol lies in the range of 6 to 53 bps. Optical communication cannot be used for long-range communication, where the optical communication range in our system is in the region of a few 10 s of cm. The optical communication range is a function of both the intensity of the RGB LED and the sensitivity of the optical sensor. In field conditions, the amount of ambient light intensity has an impact on the optical communication system.

The Limpet II possesses an IR data transceiver module. The module supports IrDA communication protocol with data rates up to 115,200 bits per second and a transmission range of up to 8 m. The module consists of an IR emitter, a PIN photodiode, and a low-power CMOS control IC. In this work, we use the IR transceiver for robot-to-computer communication, where the data from the Limpet II and the sensors can be sent via IR to the computer.

Limpet II battery life

We calculated the minimum battery life by assuming the Limpet II has all the sensors, RGB LED, microcontroller, IR communication, and EMM continuously on. The Limpet II will consume $\sim$1060 mA (communication consumes 10 mA, RGB LED consumes 20 mA, sensors consume 23 mA, microcontroller consumes 7 mA, EMM consumes 1000 mA), which allows for a battery life of about 26 min. We calculated the maximum battery life by assuming the Limpet II is in sleep mode, where it consumes an average current of 0.1 mA. In this mode, the battery life of the Limpet II can reach about 4500 h (188 days). The other Limpet II capabilities have different current consumption levels and thus can change the battery life. For example, when the limpet II is static and adhered to a surface, the Limpet II will have a battery lifetime of 50 h. In this mode, the pressure sensor consumes 2.1 mA to achieve the closed-loop control of the adhesion system, and the microcontroller consumes 7 mA.

Supplementary References

S1. Sayed ME, Nemitz MP, Aracri S, *et al.* The Limpet: a ROS-enabled multi-sensing platform for the ORCA hub. Sensors 2018;18:3487.

S2. Light RA. Mosquitto: server and client implementation of the MQTT protocol. J Open Source Softw 2017;2:265.

S3. Sornin N, Luis M, Eirich T, *et al.* LoRaWAN Specification. LoRa Alliance. Available at: https://lora-alliance.org/resource-hub/lorawanr-specification-v11 (accessed July 18, 2020).

S4. Adelantado F, Vilajosana X, Tuset-Peiro P, *et al.* Understanding the limits of lorawan. IEEE Commun Mag 2017;55:34.

S5. CableLabs. Lora server, open-source lorawan network-server. Available at: https://www.cablelabs.com/chirpstack-the-new-open-source-lora-server (accessed July 18, 2020).
