# Supplementary material for: Limpet II: A Modular, Untethered Soft Robot
Source: Soft Robot. 2021 Jun 16;8(3):319–39. doi: 10.1089/soro.2019.0161 (PMC8236390; doi:10.1089/soro.2019.0161)
Supplement: Supplemental data [file Supp_Figs1-2.pdf]

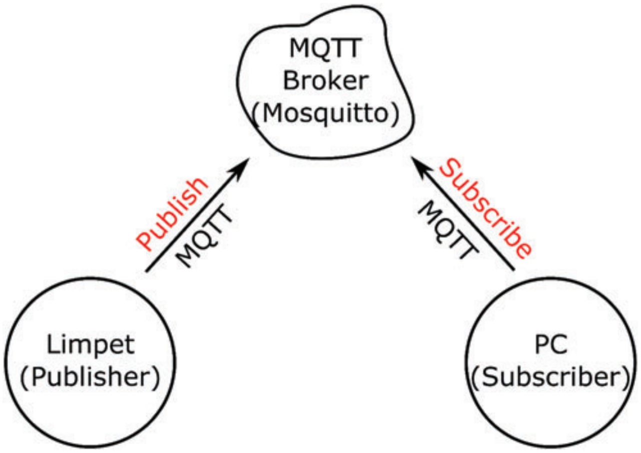

SUPPLEMENTARY FIG. S1. Architecture of the WiFi communication. In the WiFi communication strategy using the MQTT protocol, the Limpet II is the publisher, where it publishes the different sensor data, and the subscriber is the PC, where it can subscribe to the MQTT topic to receive the sensor data. The nodes (Limpet II and PC) communicate with a server, known as MQTT Broker. We use Mosquitto as the MQTT Broker. MQTT, Message Queuing Telemetry Transport.

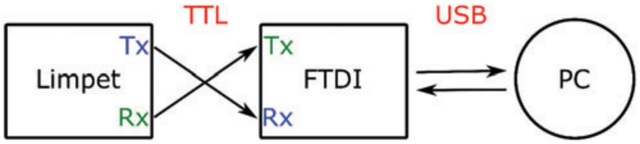

SUPPLEMENTARY FIG. S2. Architecture of the serial communication. The Limpet II sends the data serially to the computer by using the FTDI Basic Breakout, which converts the TTL communication from the Limpet II to USB signals for the PC. TTL, Transistor-Transistor Logic.
