# Supplementary material for: Limpet II: A Modular, Untethered Soft Robot
Source: Soft Robot. 2021 Jun 16;8(3):319–39. doi: 10.1089/soro.2019.0161 (PMC8236390; doi:10.1089/soro.2019.0161)
Supplement: Supplemental data [file Supp_Figs3-4.pdf]

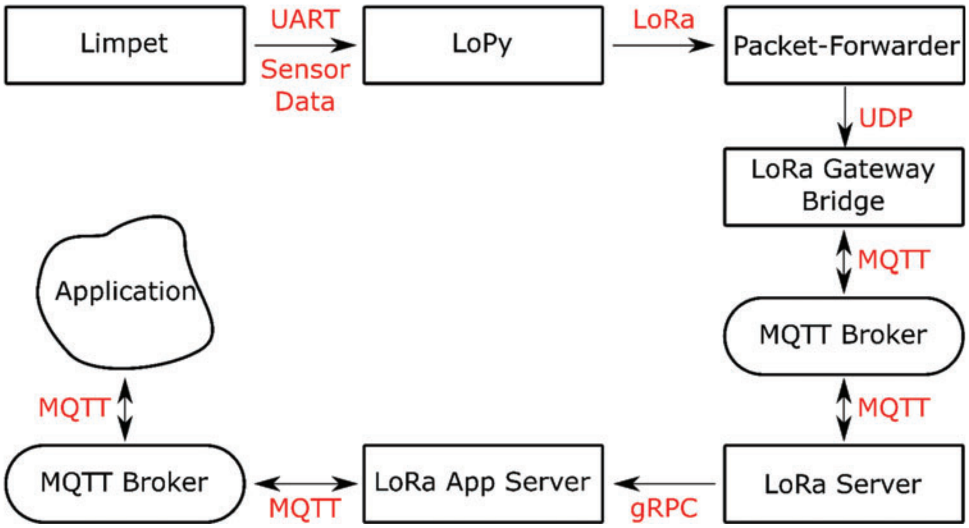

SUPPLEMENTARY FIG. S3. Architecture of the LoRaWAN communication. The Limpet II sends the sensor data packets to the LoPy node through the UART. The LoPy sends these data packets to the LoRa gateway by using the LoRaWAN wireless protocol. The gateway runs an implementation of the packet-forwarder software, which forwards the data packets to a network server (LoRa Server) by using a UDP protocol. The LoRa Gateway Bridge transfers the packet-forwarder UDP protocol into messages over MQTT. The MQTT broker routes the MQTT messages between the nodes. The MQTT messages are forwarded to the LoRa server, which provides the LoRaWAN network server component. The LoRa App Server implements a LoRaWAN application server and provides a web interface and APIs. The data packets or payload can be published from the LoRa App Server to an application by using the MQTT protocol. The application handles the application payloads sent by the Limpets. LoRaWAN, long-range wide area network; UART, Universal Asynchronous Receiver-Transmitter; UDP, User Datagram Protocol.

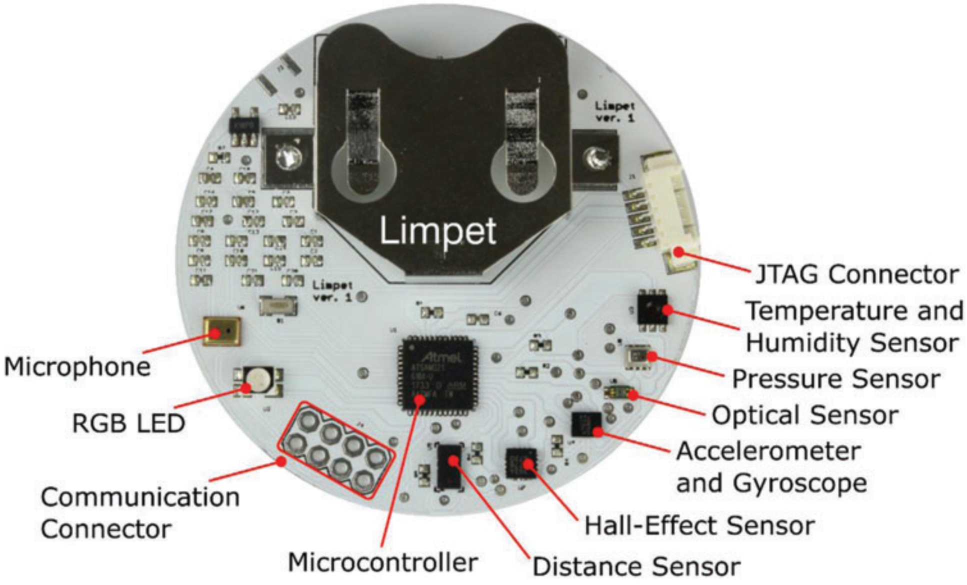

SUPPLEMENTARY FIG. S4. Labeled picture of the sensing module. A labeled picture of the sensing PCB showing the different on-board sensing modalities, microcontroller, and RGB LED. PCB, Printed Circuit Board.
