# Supplementary figures and images for: Limpet II: A Modular, Untethered Soft Robot
Source: Soft Robot. 2021 Jun 16;8(3):319–39. doi: 10.1089/soro.2019.0161 (PMC8236390; doi:10.1089/soro.2019.0161)

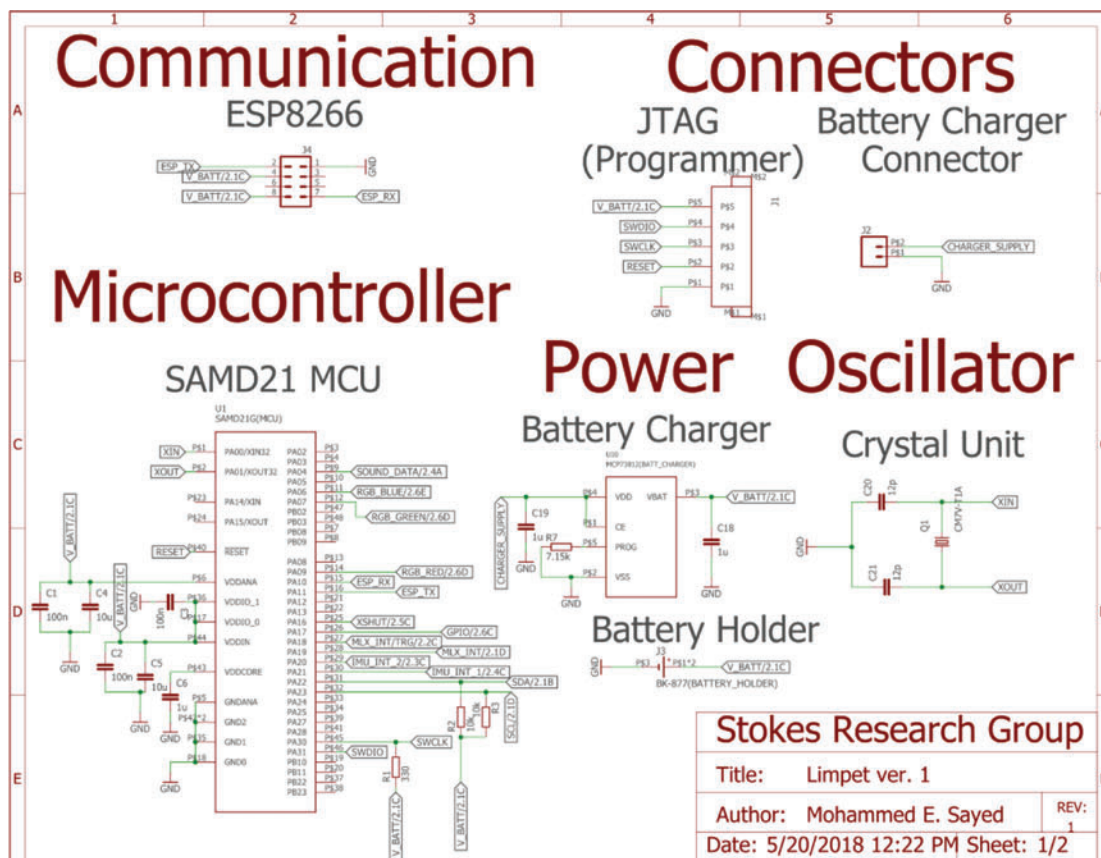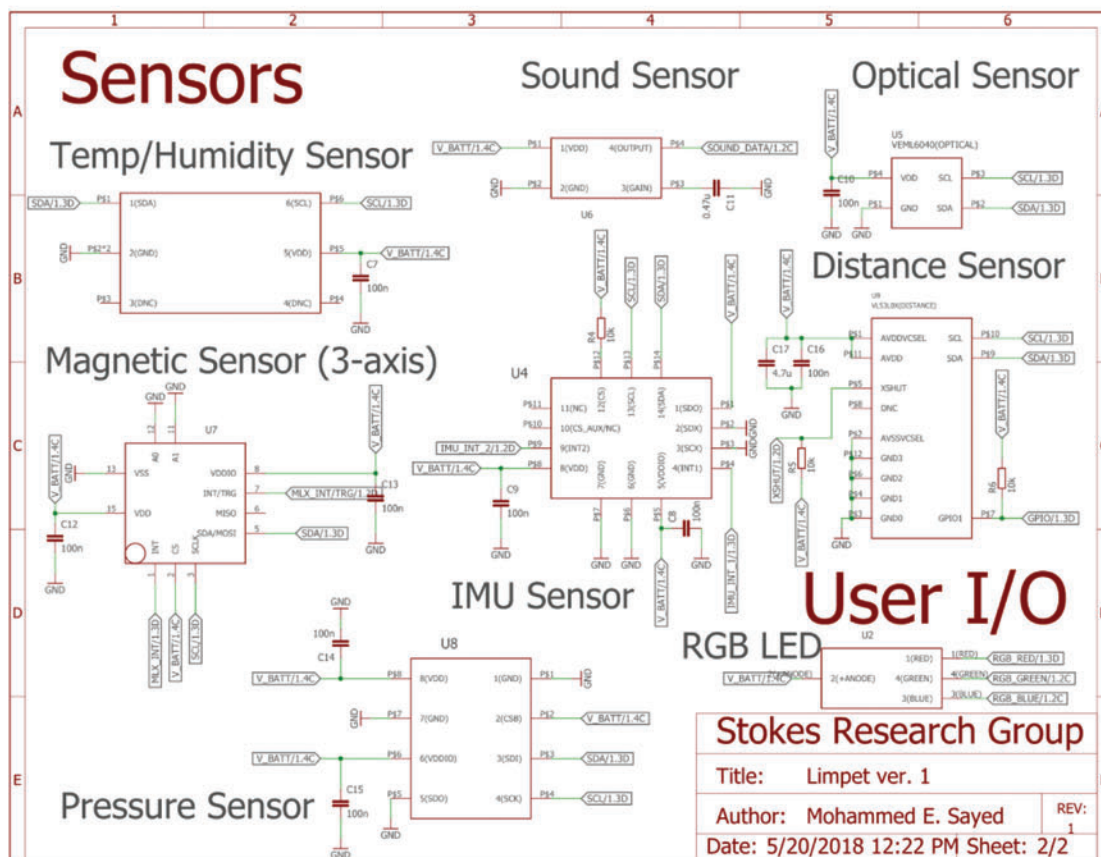

**SUPPLEMENTARY FIG. S5.** A circuit schematic of the sensing module.

Supplement: Supplemental data [file Supp_Fig5.pdf]
