# Supplementary material for: Limpet II: A Modular, Untethered Soft Robot
Source: Soft Robot. 2021 Jun 16;8(3):319–39. doi: 10.1089/soro.2019.0161 (PMC8236390; doi:10.1089/soro.2019.0161)
Supplement: Supplemental data [file Supp_Figs6-7.pdf]

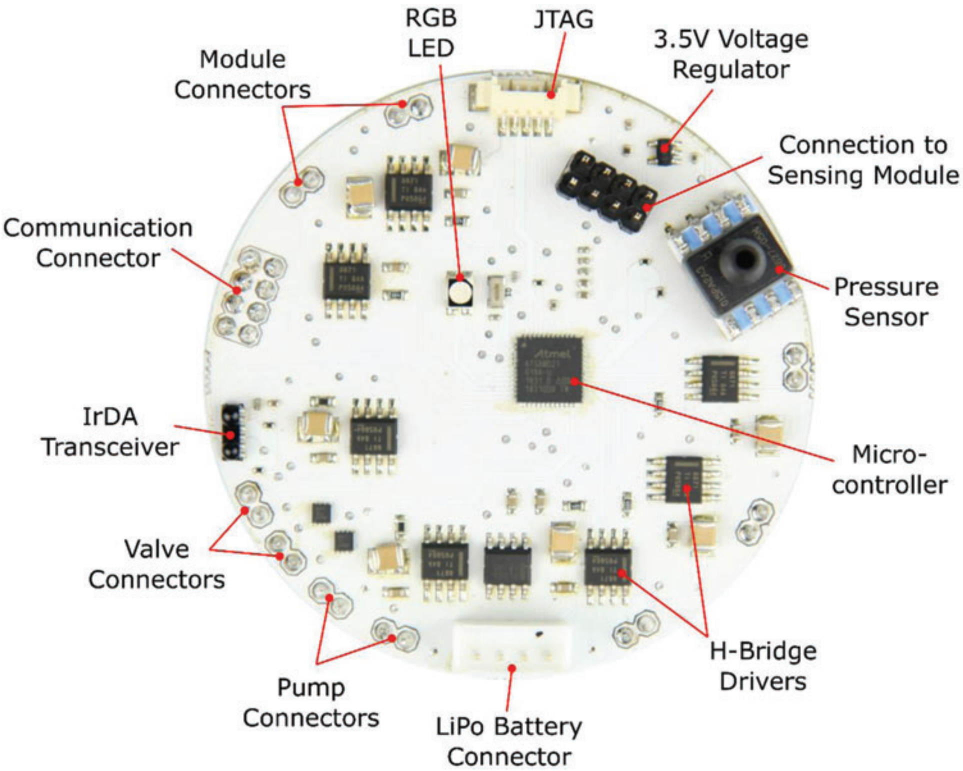

SUPPLEMENTARY FIG. S6. Labeled picture of the power driver module. A labeled picture of the power driver PCB showing the pressure sensor, h-bridges, infrared transceiver, RGB LED, board-to-board connector, communication connector, JTAG, battery connector, microcontroller, valve connector, and pump connector.

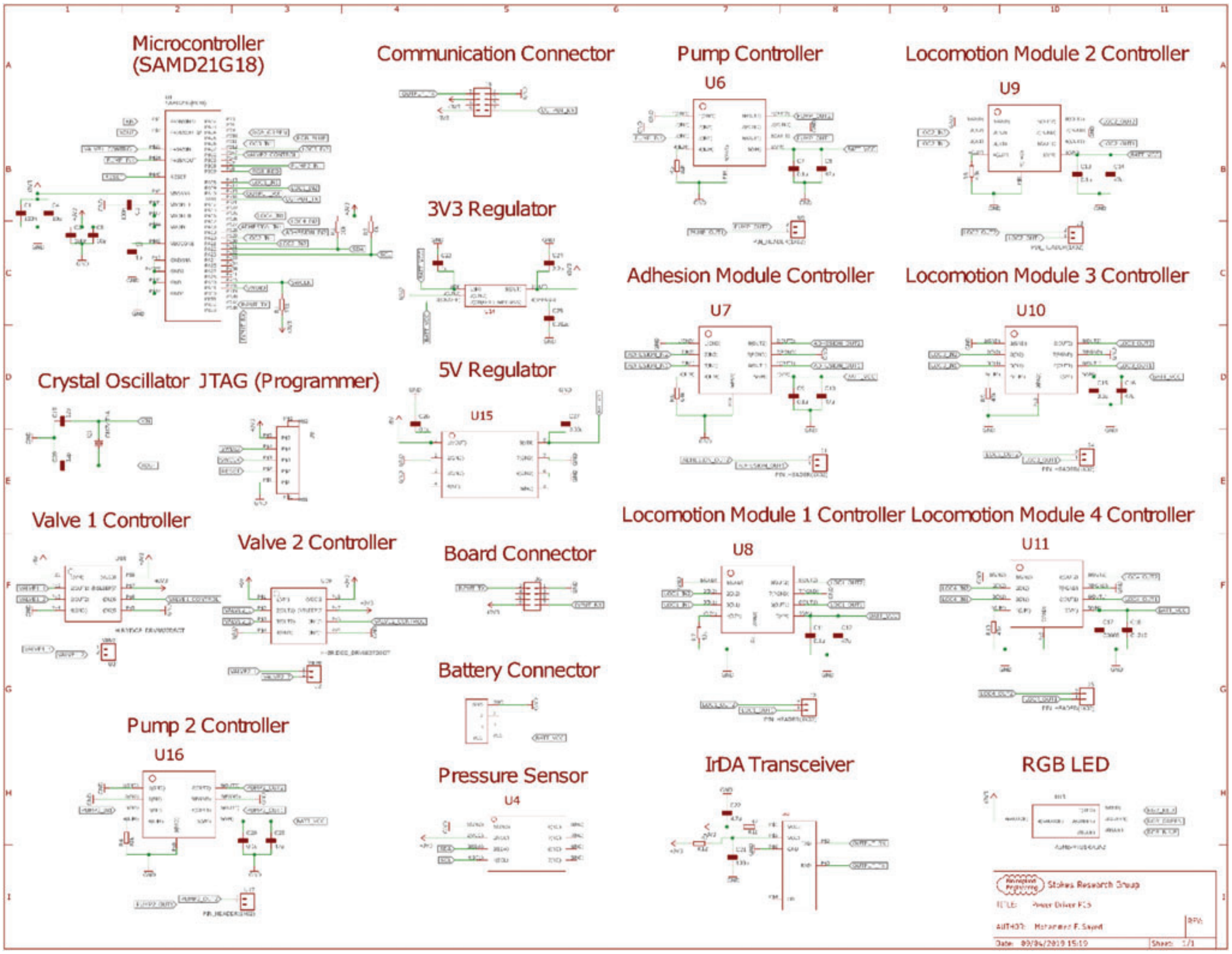

SUPPLEMENTARY FIG. S7. A circuit schematic of the power driver module.
