# Supplementary material for: Limpet II: A Modular, Untethered Soft Robot
Source: Soft Robot. 2021 Jun 16;8(3):319–39. doi: 10.1089/soro.2019.0161 (PMC8236390; doi:10.1089/soro.2019.0161)
Supplement: Supplemental data [file Supp_Figs8-9.pdf]

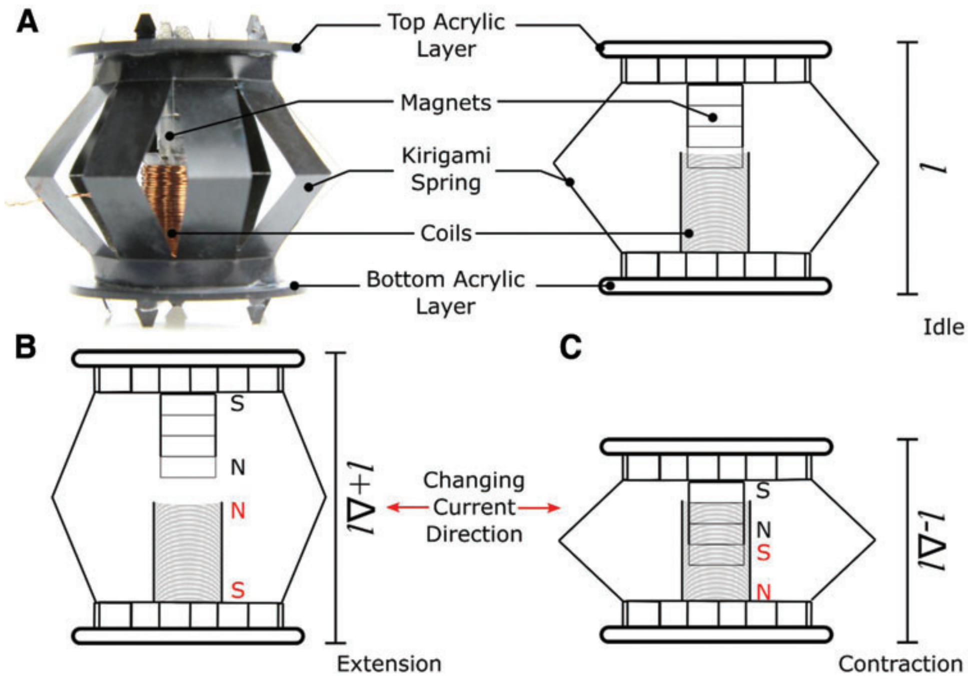

SUPPLEMENTARY FIG. S8. Sketch of the EMM and the actuation mechanism. **(A)** A labeled picture and sketch of the EMM showing all its components, and a sketch of the actuation mechanism. **(B)** Sketch of the EMM in extension mode. **(C)** Sketch of the EMM in contraction mode. EMM, electromagnetic module.

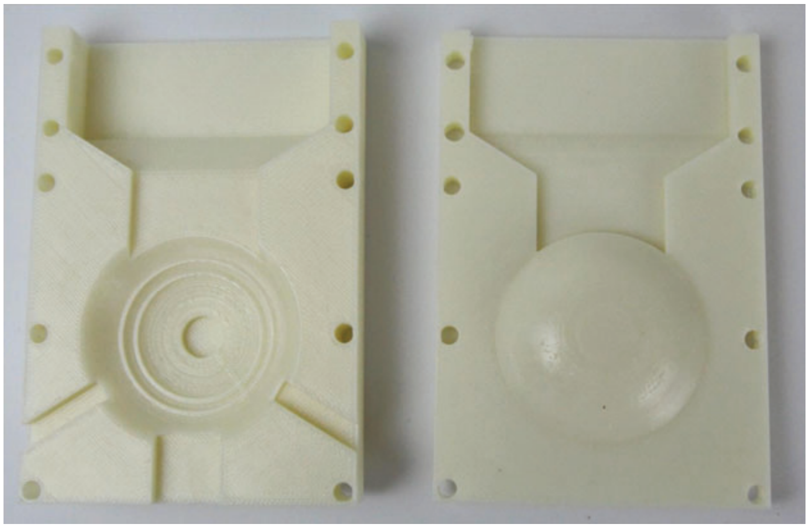

SUPPLEMENTARY FIG. S9. Mold used to fabricate the custom-designed suction cups.
