# Supplementary material for: Limpet II: A Modular, Untethered Soft Robot
Source: Soft Robot. 2021 Jun 16;8(3):319–39. doi: 10.1089/soro.2019.0161 (PMC8236390; doi:10.1089/soro.2019.0161)
Supplement: Supplemental data [file Supp_Fig11.pdf]

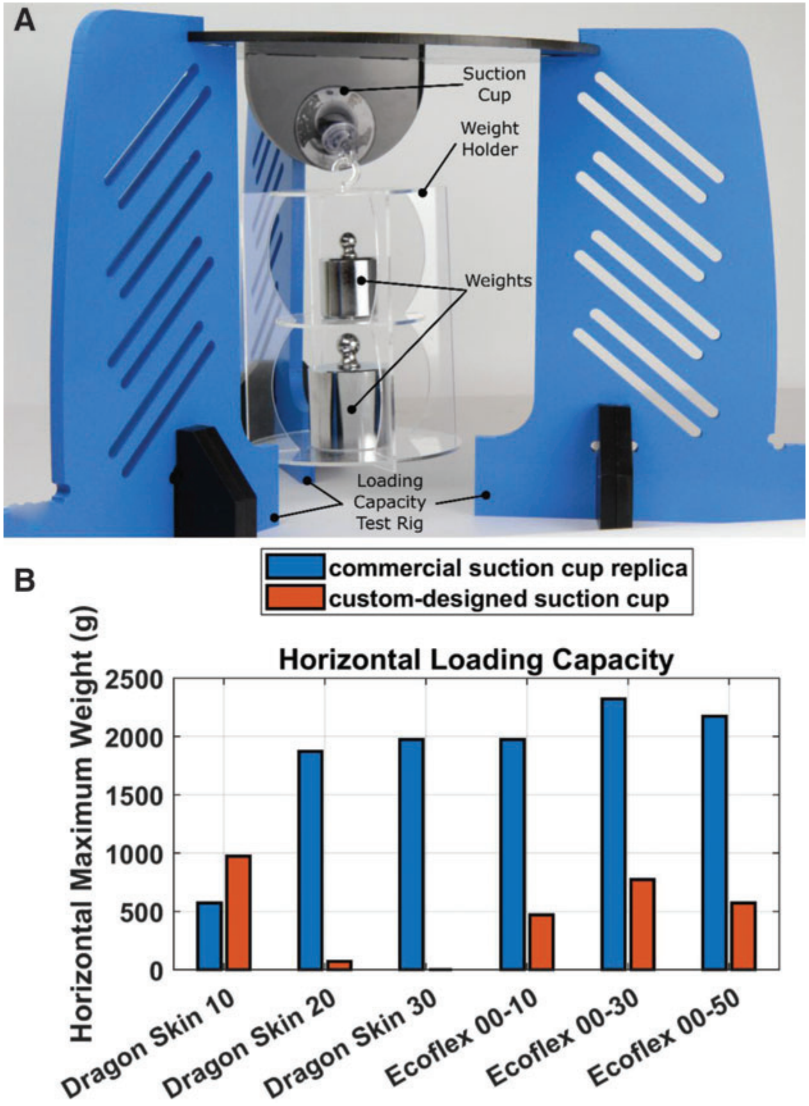

SUPPLEMENTARY FIG. S11. Horizontal loading capacity of the suction cups. **(A)** A labeled picture of the experimental setup used to test the horizontal loading capacity of suction cups. In this experiment, we fabricated two different groups of suction cups, custom-designed suction cups and replicas of the commercial suction cup, out of different soft materials: Dragon Skin 10, Dragon Skin 20, Dragon Skin 30, Ecoflex 00-10, Ecoflex 00-30, Ecoflex 00-50. **(B)** Results of the horizontal loading capacity experiment showing the maximum loading capacity for the suction cups fabricated out of the different soft materials.
