# Supplementary material for: Limpet II: A Modular, Untethered Soft Robot
Source: Soft Robot. 2021 Jun 16;8(3):319–39. doi: 10.1089/soro.2019.0161 (PMC8236390; doi:10.1089/soro.2019.0161)
Supplement: Supplemental data [file Supp_Figs12-13.pdf]

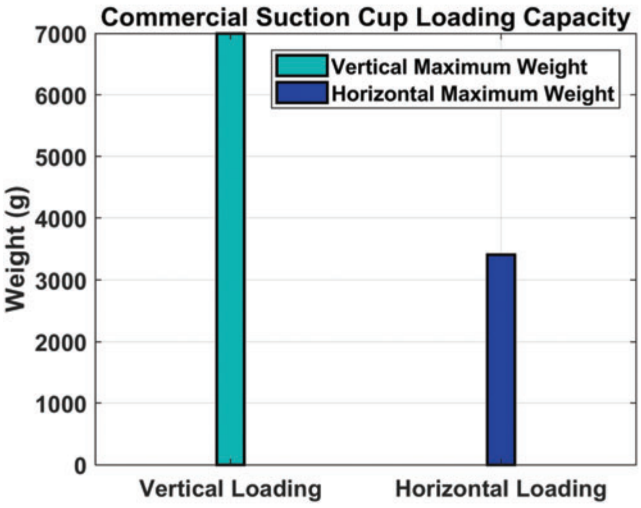

SUPPLEMENTARY FIG. S12. Horizontal and vertical loading capacity on commercial suction cups. Maximum vertical and horizontal loading capacity on the commercial suction cup used for the Limpet II. The graphs show the loading capacity of the suction cup before we make any changes to it.

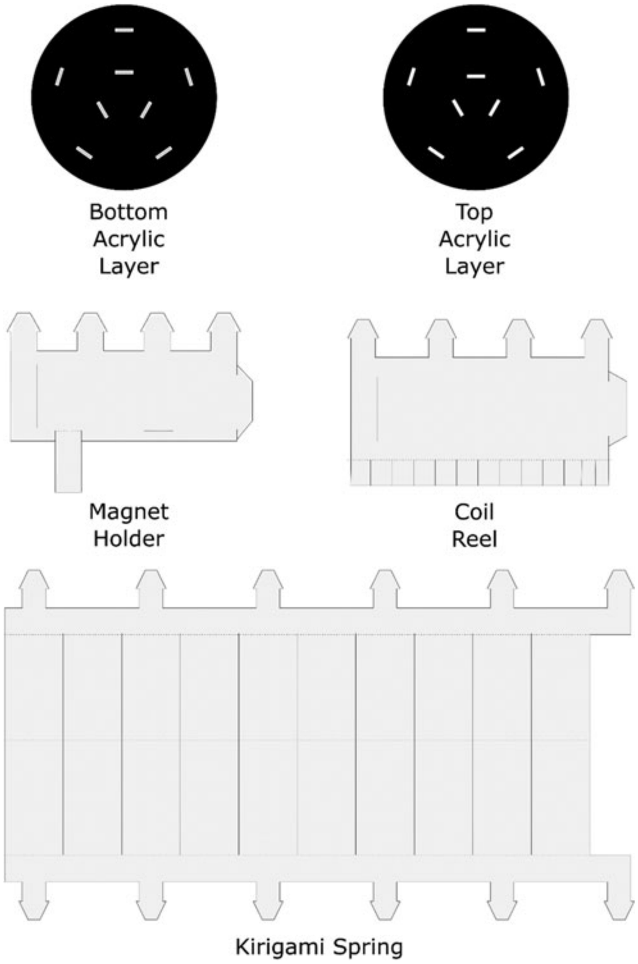

SUPPLEMENTARY FIG. S13. Two-dimensional patterns of the EMM components. Two-dimensional design of the top acrylic layer, bottom acrylic layer, magnet holder, coil reel, and kirigami spring of the EMM.
