# Supplementary material for: Limpet II: A Modular, Untethered Soft Robot
Source: Soft Robot. 2021 Jun 16;8(3):319–39. doi: 10.1089/soro.2019.0161 (PMC8236390; doi:10.1089/soro.2019.0161)
Supplement: Supplemental data [file Supp_Figs14-15.pdf]

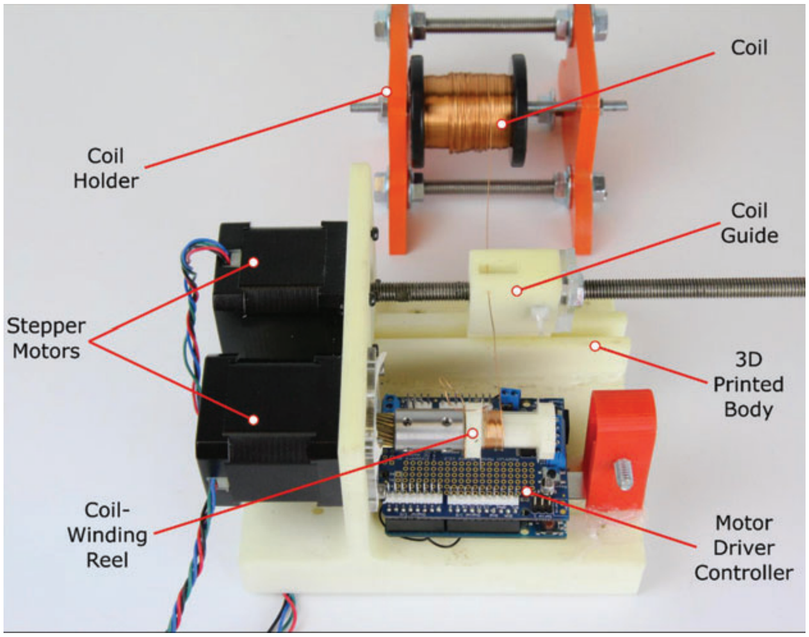

SUPPLEMENTARY FIG. S14. Custom-built coil-winding machine. A labeled picture of the custom-built coil-winding machine used to produce the coils in the EMMs.

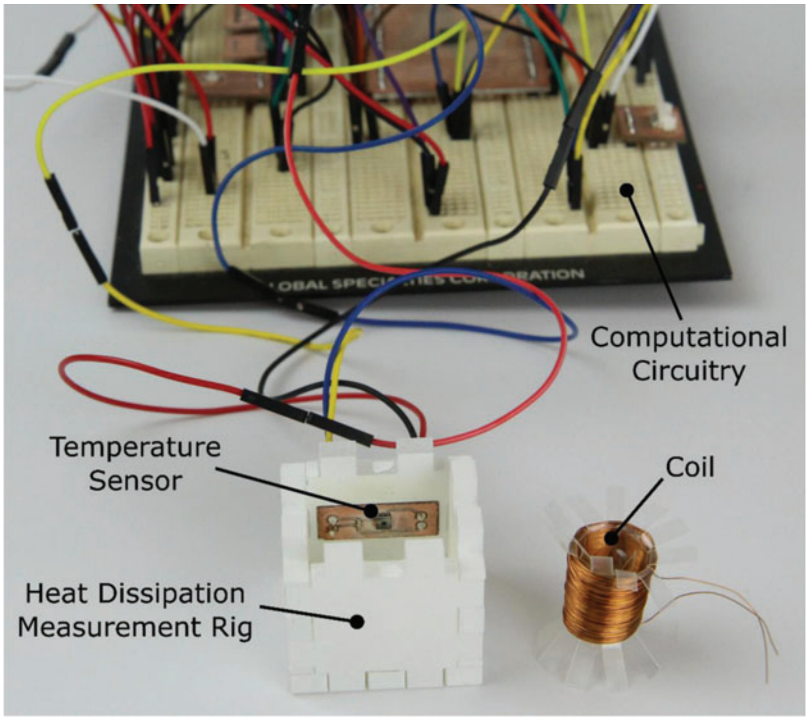

SUPPLEMENTARY FIG. S15. Experimental setup for heat dissipation measurements for different coils thickness. A picture of the experimental setup we used to analyze the effect of the coil wire thickness on the joule heating in the coils for different currents fed into the coil. We placed the coils in a custom-designed *white* acrylic box containing a temperature sensor. The temperature sensor is connected to a computational circuit consisting of a microcontroller and bypass capacitors. We feed current into the coil and measure the temperature rise as a result of the current.
