# Supplementary material for: Limpet II: A Modular, Untethered Soft Robot
Source: Soft Robot. 2021 Jun 16;8(3):319–39. doi: 10.1089/soro.2019.0161 (PMC8236390; doi:10.1089/soro.2019.0161)
Supplement: Supplemental data [file Supp_Fig16.pdf]

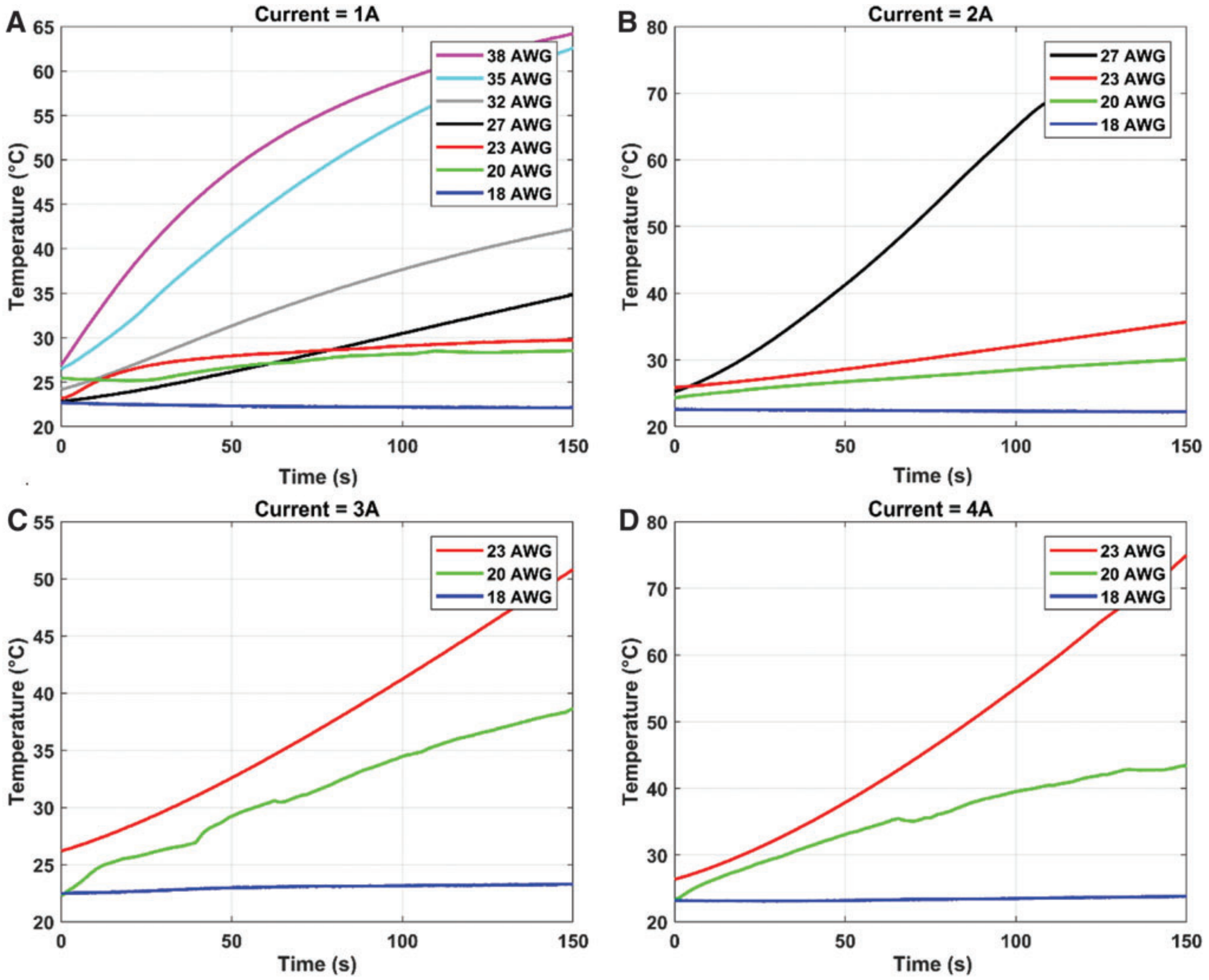

SUPPLEMENTARY FIG. S16. Experimental results of the heat dissipation experiment. Results of the heat dissipation experiments showing the rise in temperature due to different currents for the different coil thicknesses. These measurements show that the amount of current drawn by the coils is proportional to the rate of temperature rise. We analyzed the temperature rise of the coils for a period of 150 s each. Each graph shows the rise in temperature over time for each of the coils at a different current level **(A)** 1A, **(B)** 2A, **(C)** 3A, **(D)** 4A.
