# Supplementary material for: Limpet II: A Modular, Untethered Soft Robot
Source: Soft Robot. 2021 Jun 16;8(3):319–39. doi: 10.1089/soro.2019.0161 (PMC8236390; doi:10.1089/soro.2019.0161)
Supplement: Supplemental data [file Supp_Figs17-18.pdf]

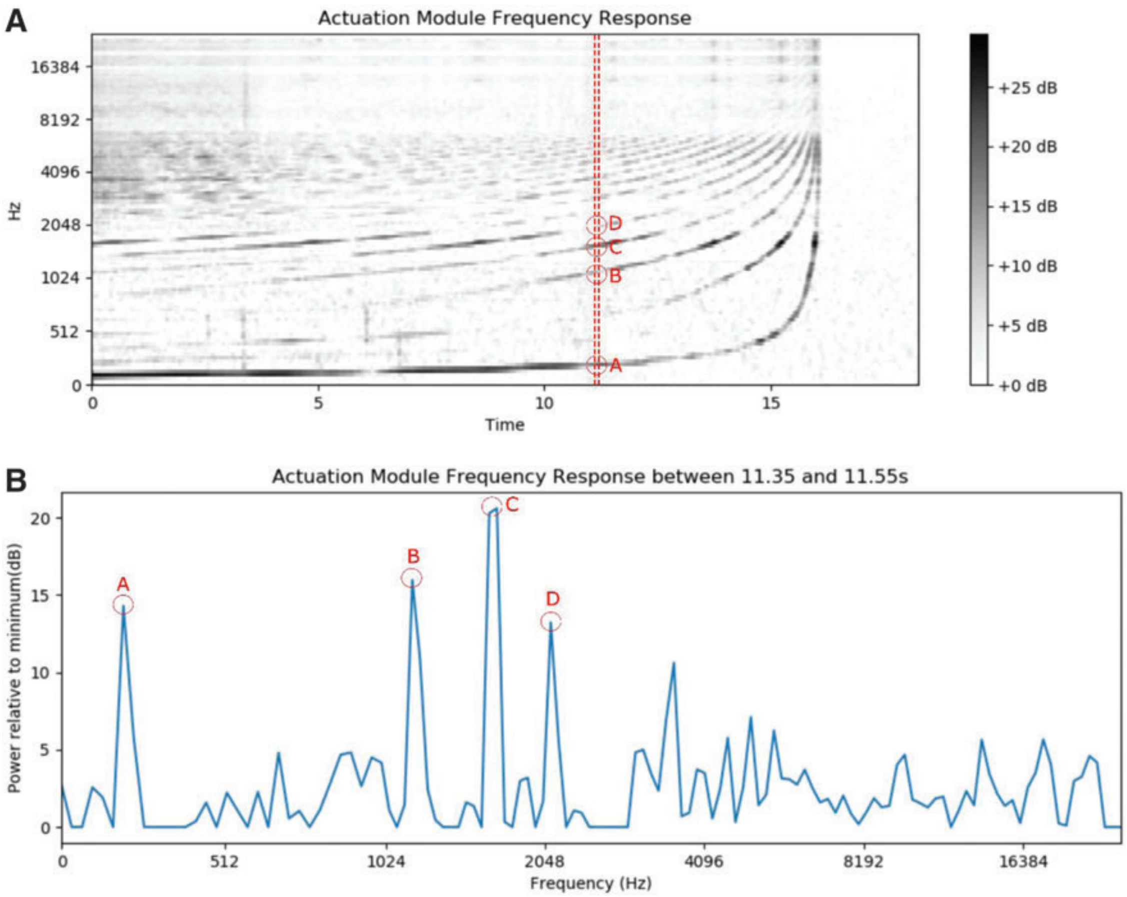

SUPPLEMENTARY FIG. S17. Sound spectrogram of the EMM at audible frequency levels. **(A)** A Mel-Spectrogram of the audio from seconds 24 to 42 of Video S2. The frequency axis is linear under 1000 Hz and is in log base 2 scale above 1000 Hz. **(B)** A vertical slice of the Mel spectrogram representing a Mel-Scaled FFT of the audio from 11.35 to 11.55 s of Video S2. FFT, fast Fourier transform.

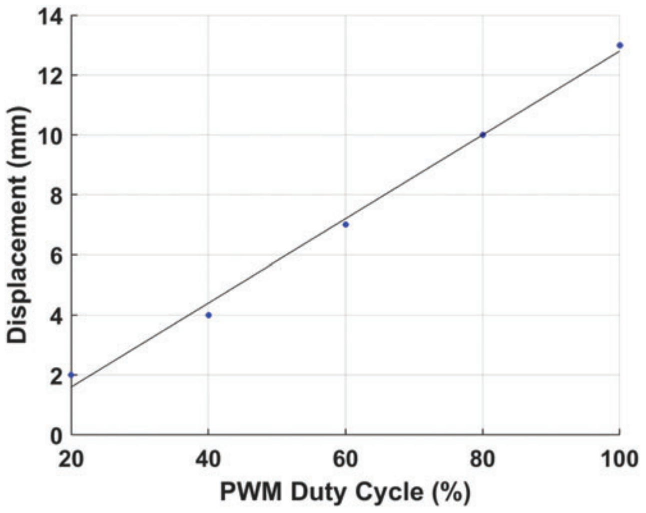

SUPPLEMENTARY FIG. S18. PWM experiment results. Results of the PWM test of the EMM showing the change in height of the EMM against the PWM duty cycle. PWM, pulse-width modulating.
