# Supplementary material for: Limpet II: A Modular, Untethered Soft Robot
Source: Soft Robot. 2021 Jun 16;8(3):319–39. doi: 10.1089/soro.2019.0161 (PMC8236390; doi:10.1089/soro.2019.0161)
Supplement: Supplemental data [file Supp_Fig19.pdf]

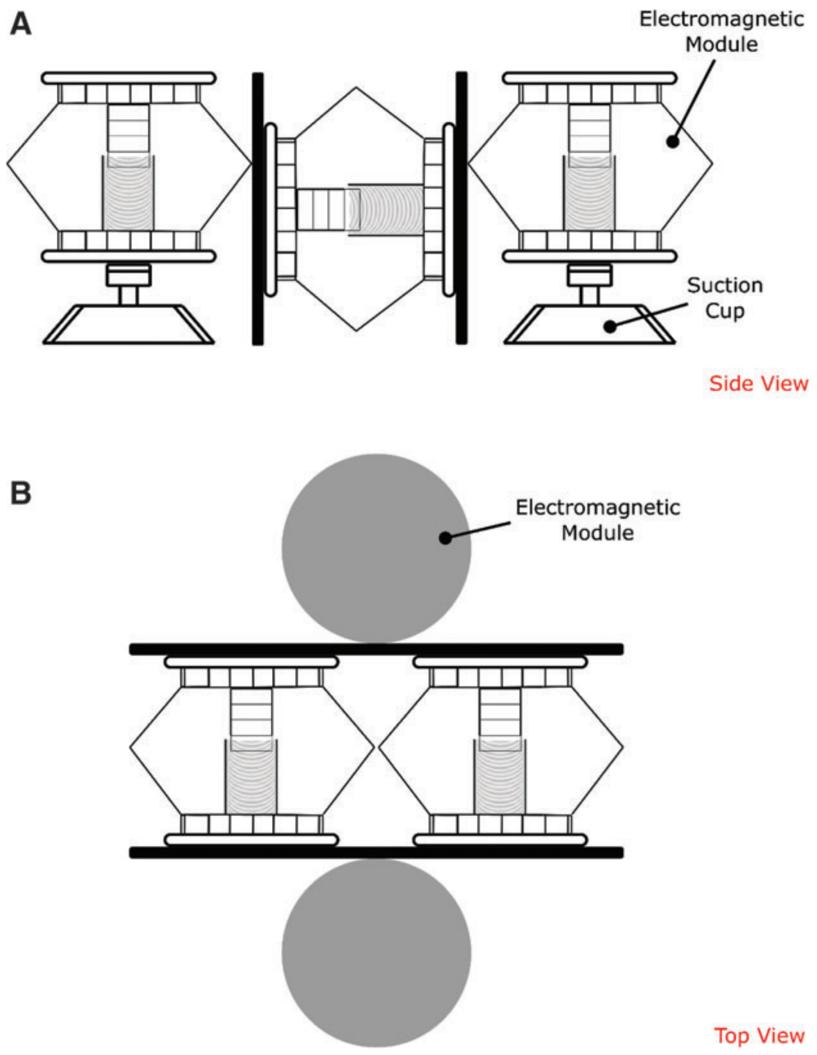

SUPPLEMENTARY FIG. S19. Configuration of the Limpet II for climbing. **(A)** Side view and **(B)** top view of the configuration used for the Limpet II to achieve climbing on a surface.
